# Podocyte Pathogenic Bone Morphogenetic Protein‐2 Pathway and Immune Cell Behaviors in Primary Membranous Nephropathy

**DOI:** 10.1002/advs.202404151

**Published:** 2024-05-24

**Authors:** Anxiang Cai, Yiwei Meng, Hang Zhou, Hong Cai, Xinghua Shao, Qin Wang, Yao Xu, Yin Zhou, Wenyan Zhou, Luonan Chen, Shan Mou

**Affiliations:** ^1^ Department of Nephrology, Ren Ji Hospital, School of Medicine Shanghai Jiao Tong University Shanghai 200127 China; ^2^ Key Laboratory of Systems Biology, Shanghai Institute of Biochemistry and Cell Biology, Center for Excellence in Molecular Cell Science Chinese Academy of Sciences Shanghai 200031 China; ^3^ Institute of Molecular Medicine, Ren Ji Hospital, School of Medicine Shanghai Jiao Tong University Shanghai 200127 China; ^4^ Key Laboratory of Systems Health Science of Zhejiang Province, Hangzhou Institute for Advanced Study University of Chinese Academy of Sciences Hangzhou 310024 China; ^5^ School of Life Science and Technology Shanghai Tech University Shanghai 201210 China

**Keywords:** bone morphogenetic protein 2, chronic kidney disease, podocyte, primary membranous nephropathy, proteinuria, renal immune cell, single‐cell RNA sequencing

## Abstract

Primary membranous nephropathy (PMN) is one of the leading causes of end‐stage renal disease, and the most frequent cause of massive proteinuria in nondiabetic adults, resulting in fatal complications. However, the underlying pathomechanisms of PMN remain largely unclear. Here, single‐cell RNA sequencing is employed to analyze kidney biopsies from eleven PMN patients and seven healthy subjects. Profiling 44 060 cells from patients allowed us to characterize the cellular composition and cell‐type‐specific gene expression in the PMN kidney. The complement‐induced BMP2/pSMAD1/COL4 pathway is identified as the pathogenic pathway in podocytes, bridging two key events, i.e., complement system activation and glomerular basement membrane thickening in PMN. Augmented infiltration and activation of myeloid leukocytes and B lymphocytes are found, profiling delicate crosstalk of immune cells in PMN kidneys. Overall, these results provide valuable insights into the roles of podocytes and immune cells in PMN, and comprehensive resources toward the complete understanding of PMN pathophysiology.

## Graphical abstract

This study established the largest single‐cell RNA database of PMN kidney biopsy samples from eleven PMN patients and seven control subjects, and characterized the cellular composition and cell‐type‐specific gene expression in PMN. The complement‐induced BMP2/pSMAD1/COL4 pathway as the pathogenic pathway in podocytes is identified, bridging complement system activation and GBM thickening in PMN. It is also found that augmented infiltration and activation of myeloid leukocytes and B lymphocytes in PMN kidneys.

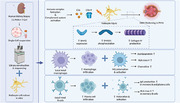

## 1 Introduction

Chronic kidney disease (CKD) has one of the most substantial global tolls on human health, and proteinuria is one of the key clinical features of CKD.^[^ ^1^ ^]^ Massive proteinuria (>40 mg m^2^ per hour) typically results in nephrotic syndrome, which leads to fatal infection, thromboembolism, acute kidney injury, and end‐stage renal disease (ESRD).^[^ ^2^ ^]^ Glomerular diseases are the most common cause of proteinuria, among which primary membranous nephropathy (PMN) represents the most frequent pathological type of nephrotic syndrome in nondiabetic adults, with a kidney biopsy rate of 20%–37%.^[^ ^3^ ^]^ PMN is an autoimmune glomerulopathy, characterized by pathological thickening of the glomerular basement membrane (GBM) and augmented urinary protein excretion.^[^ ^3a^ ^]^

Podocytes, as the major targets of PMN, play a central role in its pathogenesis. Initial histopathological recognition of PMN is GBM thickening and the formation of subepithelial “spikes”.^[^ ^4^ ^]^ The discovery of M‐type phospholipase A2 receptor (PLA2R) as the most frequent target antigen enabled further understanding of the pathomechanisms of PMN.^[^ ^5^ ^]^ PLA2R is the most common autoantigen of PMN expressed on the human podocyte surface.^[^ ^4^ ^]^ In PMN, the abnormally activated immune system produces IgG4 autoantibodies targeting podocyte surface autoantigens, which are deposited in the glomerular subepithelial area and trigger complement system activation, ultimately resulting in podocyte injury and impairment of the glomerular filtration barrier.^[^ ^3^, ^4^, ^5^ ^]^ However, the underlying pathways that regulate immune disorder, podocyte injury, and GBM thickening remain largely unknown, and deserve in‐depth investigations.

Single‐cell RNA sequencing (scRNA‐seq) represents a promising method to reveal cell‐type‐specific pathogenic pathways. Nevertheless, such applications are limited in studying glomerular diseases by the small amounts of tissue that can be obtained by core needle kidney biopsy.^[^ ^6^ ^]^ To address this problem, we performed large‐scale scRNA‐seq with kidney biopsy samples from eleven PMN patients and seven control subjects, which allowed us to present a comprehensive transcriptomic profile of the diseased kidney in PMN. In combination with immunostaining of kidney biopsy sections and cell culture of immortalized mouse podocytes, we demonstrate the role of the complement‐induced BMP2/pSMAD1/COL4 pathway, and reveal renal B‐cell and macrophage activation in PMN, thereby providing valuable resources for the mechanistic understanding of PMN and new insights into its therapeutic targets.

## 2 Results

### 2.1 Single‐Cell RNA Sequencing Profile of Kidney Cell Clusters

**Figure 1:**
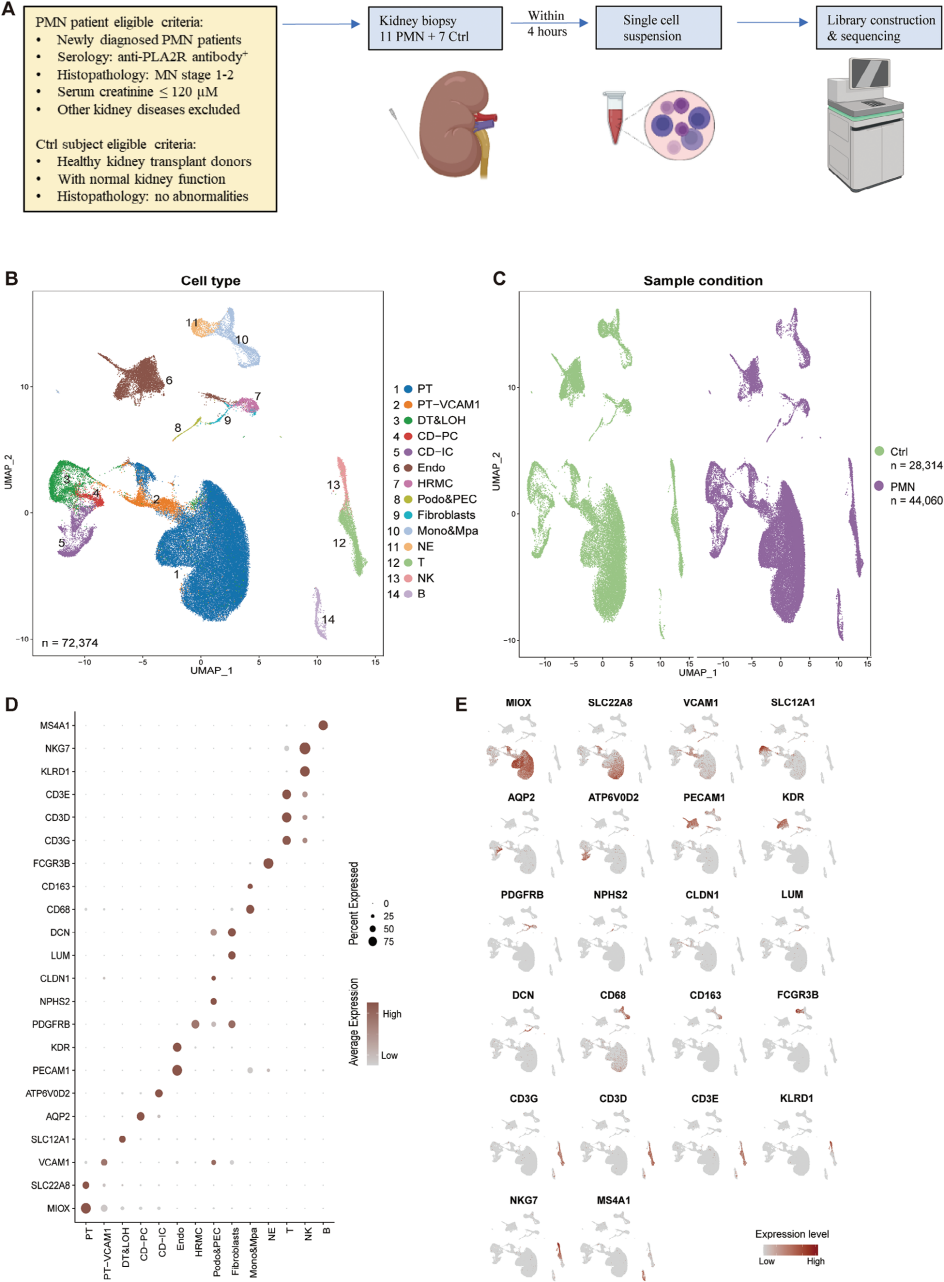
Single‐cell RNA sequencing profile of kidney cell clusters. A) Schematic illustration of the study design, eligibility criteria, and sample collection and processing strategies. B) Uniform manifold approximation and projection (UMAP) indicating a total of 72 374 cells color‐coded by different cell types. Endo, endothelial cells; Podo, podocytes; PEC, glomerular parietal epithelial cells; HRMC, human renal mesangial cells; Fibro, fibroblasts; B, B lymphocytes; Mono, monocytes; Mpa, macrophages; NE, neutrophils; T, T lymphocytes; NK, natural killer cells; PT, proximal tubule; LOH, loop of Henle; DT, distal tubule; CD‐PC, collecting duct principal cells; CD‐IC, collecting duct intercalated cells. C) UMAP indicating cell cluster distribution between PMN patients and control subjects. D) Dot plot showing the expression of marker genes in different cell types. E) Respective UMAP indicating the distribution of marker genes. See also Figure S1 (Supporting Information).

Diseased kidney biopsy samples were obtained from eleven newly diagnosed PMN patients with positive anti‐PLA2R autoantibody in serum, histopathological diagnosis of MN stage I‐II, and without other kidney diseases; control (Ctrl) samples were obtained from seven subjects with normal kidney function and without histopathological abnormalities in selected kidney biopsy tissue (**Figure** **1**A). The proteinuria levels of patients ranged from 773.8 mg/24 h to 11289.2 mg/24 h, and there was no statistically significant difference in age (58.6 ± 8.5 vs 53.3 ± 11.7 years, *P* = 0.3060), serum creatinine (67.1 ± 17.3 vs 67.7 ± 8.2 µM, *P* = 0.9341), or estimated glomerular filtration rate (89.7 ± 18.8 vs 102.0 ± 10.1 ml min/1.73 m^2^, *P* = 0.1530) between PMN and Ctrl subjects, but several patients were at CKD stage 2 (**Table** **1**). After quality control (Figure S1A, Supporting Information), a total of 72374 cells were retained for subsequent analysis (Figure 1B), of which 44060 cells (60.88%) were from patients and 28314 cells (39.12%) were from Ctrls (Figure 1C). Fourteen clusters of kidney cells within five major categories were identified by graph‐based clustering (Figure 1; Figure S1B, Supporting Information) and annotated according to known marker genes (Table S1, Supporting Information): endothelial cells (Endos), podocytes and glomerular parietal epithelial cells (Podos&PECs), human renal mesangial cells (HRMCs) and fibroblasts (Fibros) were glomerular and interstitial cells; B lymphocytes (B) represented a unique category; monocytes together with macrophages (Monos&Mpas) and neutrophils (NEs) were myeloid immunocytes; T cells (T) and natural killer cells (NKs) were divided into a category; and tubular cells were composed of the proximal tubule (PT), distal tubule with the loop of Henle (DT&LOH), collecting duct‐principal cells (CD‐PCs) and collecting duct‐intercalated cells (CD‐ICs) (Figure 1B–E). Of note, due to the complexity of renal cell types and the similarity of some cell compositions, the resolution of UMAP representation after first clustering was relatively lower, and several cell clusters could hardly be separated from other clusters (e.g., renal interstitial cells and glomerular cells). Therefore, a subclustering was performed each time we analyzed a specific cluster of renal cells.

**Table 1:** Clinical features of PMN patients and control subjects.

ID	Gender	Age (years)	Pathological Diagnosis	24 h Proteinuria (mg)	Serum Creatinine (µM)	eGFR (mL min^−1^/1.73m^2^)	CKD Stage
PMN_1	Female	53	MN Stage I	2976.4	51	106	1
PMN_2	Male	58	MN Stage I	6971.7	99	72	2
PMN_3	Female	72	MN Stage I	3368.5	83	61	2
PMN_4	Female	55	MN Stage I	773.8	45	109	1
PMN_5	Female	40	MN Stage II	4735.4	44	122	1
PMN_6	Female	56	MN Stage II	11 289.2	88	64	2
PMN_7	Female	56	MN Stage II	1965.7	75	77	2
PMN_8	Male	60	MN Stage II	2235.0	63	102	1
PMN_9	Female	70	MN Stage II	1063.4	55	92	1
PMN_10	Female	67	MN Stage II	3186.4	61	85	2
PMN_11	Male	58	MN Stage II	2475.0	74	97	1
Ctrl_1	Male	46	/	/	67	112	/
Ctrl_2	Male	57	/	/	71	101	/
Ctrl_3	Male	67	/	/	74	93	/
Ctrl_4	Female	57	/	/	56	91	/
Ctrl_5	Male	45	/	/	77	106	/
Ctrl_6	Male	68	/	/	74	92	/
Ctrl_7	Female	33	/	/	55	119	/

^a)^ eGFR, estimated glomerular filtration rate;

^b)^ CKD, chronic kidney disease.

Considering the anatomical and sampling differences in individuals, the proportions of samples were not uniform in some cell clusters, which may lead to the domination of results by a subject with a substantial number of cells. Thus, a random subsampling method was utilized to test the consistency of alternations in patients (Figure S1C,D, Supporting Information). We confirmed that our observations were common in most PMN patients, and suitable for the analysis of PMN pathophysiology.

### 2.2 Renal Glomerular and Interstitial Cell Alterations in PMN

**Figure 2:**
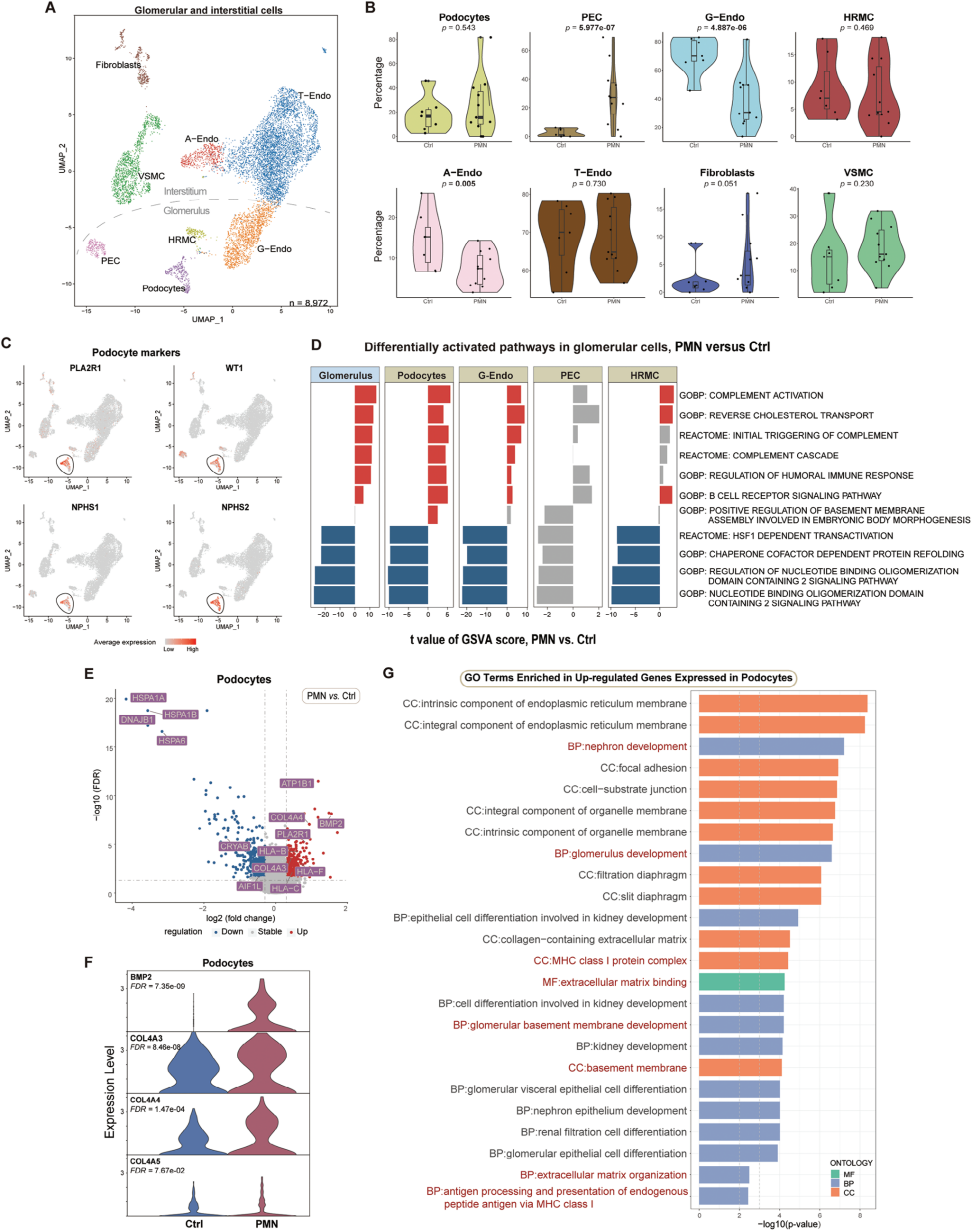
Renal glomerular cell alterations in PMN. A) UMAP indicating subclustered glomerular and interstitial cells color‐coded by different cell types. B) Respective cell percentages of renal glomerular and interstitial cells in PMN versus Ctrl. C) Expression distribution of podocyte marker genes. D) Major upregulated (red) and downregulated (blue) pathways of all glomerular cells in PMN versus Ctrl, according to GSVA. E) Volcano plot showing major upregulated (red) and downregulated (blue) genes in podocytes. F) Violin plot showing the expression levels of *BMP2*, *COL4A3*, *COL4A4*, and *COL4A5* in podocytes. G) Gene Ontology (GO) analysis presenting major upregulated pathways of podocytes in terms of biological process (BP), cellular component (CC), and molecular function (MF), with pathways of interest marked red. See also Figures S2 and S3 (Supporting Information).

To acquire a more precise glomerular and interstitial cell type division, we subclustered the glomerular and interstitial category and annotated eight clusters as Podos, PECs, *EHD3^+^* glomerular endothelial cells (G‐Endos),^[^ ^7^ ^]^ *SEMA3G^+^* arterial endothelial cells (A‐Endos),^[^ ^8^ ^]^ peritubular endothelial cells together with ascending vasa recta and descending vasa recta (T‐Endos), HRMCs, vascular smooth muscle cells (VSMCs), and Fibros (**Figure** **2**A–C; Figure S2A–D, Supporting Information). Podos, PECs, HRMCs, and G‐Endos were identified as glomerular cells; while A‐Endos, T‐Endos, VSMCs, and Fibros as interstitial cells.

We first dived into the glomerulus, the major region of PMN lesions.^[^ ^3a^ ^]^ There was no significant difference in the proportion of podocytes (Figure 2B,C, Tables S2–S4, Supporting Information), which corresponded to our hypothesis and former investigations that attacks from complement components cause injury rather than enormous death of podocytes.^[^ ^3a^ ^]^ Although PECs and G‐Endos seemed to show changes in percentages between Ctrl and PMN (Figure 2B), G‐Endos, PECs, and HRMCs displayed comparable, non‐specific gene expression profiles, such as elevated ribosomal synthesis of proteins and aerobic respiration activities, and only podocytes presented distinct transcriptomic profiles (Figure S2E, Supporting Information). Correspondingly, differentially activated pathways in all glomerular cells of PMN versus Ctrl were identified based on Gene Set Variation Analysis (GSVA),^[^ ^9^ ^]^ which indicated enhanced lipid transport, complement system activation, and humoral immune response in Podos, G‐Endos, and HRMCs; while synthesis of GBM component extracellular matrix (ECM) was uniquely activated in podocytes (Figure 2D). In contrast, downregulated protein refolding, heat shock proteins (HSP), and NOD2 signaling pathways were detected in the PMN glomerulus (Figure 2D).

The differentially expressed genes (DEGs) of podocytes were further explored to interpret the mechanism that governs podocyte injury. Identification of podocytes (N = 295) was confirmed via four recognized marker genes: *PLA2R1*, *WT1*, *NPHS1*, and *NPHS2* (Figure 2C). Among the top upregulated genes of podocytes in PMN, *bone morphogenetic protein 2* (*BMP2*), a well‐characterized bone development inducer belonging to the transforming growth factor beta superfamily,^[^ ^10^ ^]^ presented higher expression together with its possible target genes, two major ECM components of GBM,^[^ ^11^ ^]^ *COL4A3* and *COL4A4* (Figure 2E and F; Figure S3A, Supporting Information). These DEGs in PMN podocytes were noticed as they enriched Gene Ontology (GO) terms related to ECM synthesis of GBM components (Figure 2G). As for the specificity of these DEGs to PMN podocytes, we analyzed their expression levels in other glomerular cells in PMN (Figure S2F, Supporting Information), and data from two previous studies on another common proteinuric disease IgA nephropathy^[^ ^12^ ^]^ (HRA000342 with renal biopsies of 13 IgAN patients and normal tissues from 6 controls, and GSE171314 with renal biopsies of 4 IgAN patients and normal tissues from 1 control). We found that *BMP2, COL4A3*, and *COL4A4* were not upregulated either in other glomerular cells in PMN or in podocytes/HRMCs in IgA nephropathy (Figure S3B, Supporting Information). Such analysis suggested the specificity of these DEGs to PMN podocytes, or at least not in IgAN.

Moreover, podocytes from PMN patients showed augmented mRNA levels of *PLA2R* and class I human leukocyte antigen (HLA), including *HLA‐A*, *HLA‐B*, and *HLA‐F* (Figure 2E), suggesting more active autoantigen presentation through major histocompatibility complex (MHC) class I (Figure 2G). For downregulated genes in PMN podocytes, we observed reduced mRNA levels of *CRYAB* and *AIF1L* (Figure 2E), which are essential for podocyte cytoskeletons, and their downregulation is associated with foot process effacement and filtration barrier impairment in PMN.^[^ ^13^ ^]^ GO enrichment revealed that downregulated pathways in PMN podocytes were enriched in molecular chaperones and protein folding (Figure S3C, Supporting Information). Considering the latent transition associated with BMP2 expression in podocytes during PMN initiation and development, our previous algorithm^[^ ^14^ ^]^ for disease early warning was employed to predict a potential tipping state of PMN podocytes, which is a key cell state to indicate the initiation or the critical transition for PMN. As a result, cell state 2 was detected as a potential tipping state among all three inferred podocyte sub‐states (Figure S3D,E, Supporting Information), and functional enrichment indicated a close relation between podocytes in cell state 2 and PMN evolution through ECM production triggered by *BMP2* expression (Figure S3F, Supporting Information).

Collectively, our data unraveled podocyte‐specific signals and behaviors in PMN, identifying BMP2 as a potential mediator molecule for podocyte ECM production and GBM thickening.

### 2.3 Complement‐Induced Podocyte BMP2/pSMAD1/COL4 Signaling In Vitro and In Vivo

**Figure 3:**
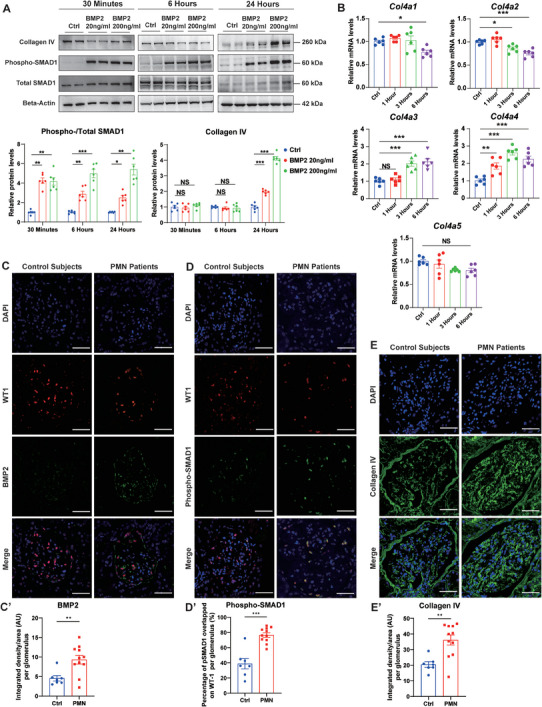
Podocyte BMP2/pSMAD1/COL4 signaling activation in vitro and in vivo. A) Western blot analysis showing the protein levels of collagen IV and phosphorylated and total SMAD1 in podocyte cell lysates after incubation with BMP2 recombinant protein for different durations and concentrations, with corresponding quantifications. Beta‐actin was used as the loading control. B) Quantitative real‐time PCR (qPCR) analysis showing podocyte mRNA levels of *Col4a1, Col4a2, Col4a3, Col4a4, and Col4a5* after incubation with 20 ng/ml BMP2 for different durations. C) Immunofluorescence analysis of DAPI (blue), WT1 (red), and BMP2 (green) expression in paraffin‐embedded kidney biopsy sections of PMN patients (upper) and control subjects (lower). D) Immunofluorescence analysis of DAPI (blue), WT1 (red), and phosphorylated SMAD1 (green) expression in paraffin‐embedded kidney biopsy sections of PMN patients (upper) and control subjects (lower). E) Immunofluorescence analysis of DAPI (blue), and collagen IV (green) expression in paraffin‐embedded kidney biopsy sections of PMN patients (upper) and control subjects (lower). C’. Quantification of BMP2 fluorescence density per glomerulus. D’. Quantification of the percentage of pSMAD1 and WT‐1 double‐positive cells per glomerulus. E’. Quantification of Collagen IV fluorescence density per glomerulus. For C’‐E’, each dot represents the average value from five random glomerular images from each patient/control. *P < 0.05; **P < 0.01; ***P < 0.001; NS, not significant. Scale bar = 50 µm. Each experiment was replicated for 5 times for A and B. N = 6 per group for A and B. For C’‐E’, N = 7 for Ctrl, N = 11 for PMN. Data are presented as mean ± SEM. One‐way ANOVA test for B, and Student's two‐tailed t‐test for C’‐E’.

To confirm our findings regarding the BMP2/COL4 signaling pathway in PMN, immortalized mouse podocytes were cultured and incubated with BMP2 recombinant protein in vitro. We detected phosphorylated SMAD1 (pSMAD1), a canonical intracellular messenger of BMP2,^[^ ^15^ ^]^ at as early as 30 min (**Figure** **3**A). Significant elevation of *Col4a3* mRNA levels was detected at 3 h post‐treatment, while that of *Col4a4* increased at 1 h post‐incubation (Figure 3B). Other collagen IV chain isoforms *Col4a1, Col4a2, and Col4a5* showed no upregulation after BMP2 treatment for 6 h (Figure 3B). Furthermore, incubation with BMP2 for 24 h increased the protein level of collagen IV, accompanied by robust SMAD1 phosphorylation (Figure 3A). In PMN kidney biopsies from patients inside (Figure 3C–E) and outside (Figure S4, Supporting Information) of our scRNA‐seq cohort, immunofluorescence revealed elevated BMP2 expression around WT1 (podocyte nuclear marker) positive cells (Figure 3C), upregulated SMAD1 phosphorylation that colocalized with WT1 (Figure 3D), and augmented collagen IV levels in GBM compared with Ctrls (Figure 3E). In combination, such results validated the BMP2/pSMAD1/COL4 pathway in podocytes both in vitro and in vivo.

**Figure 4:**
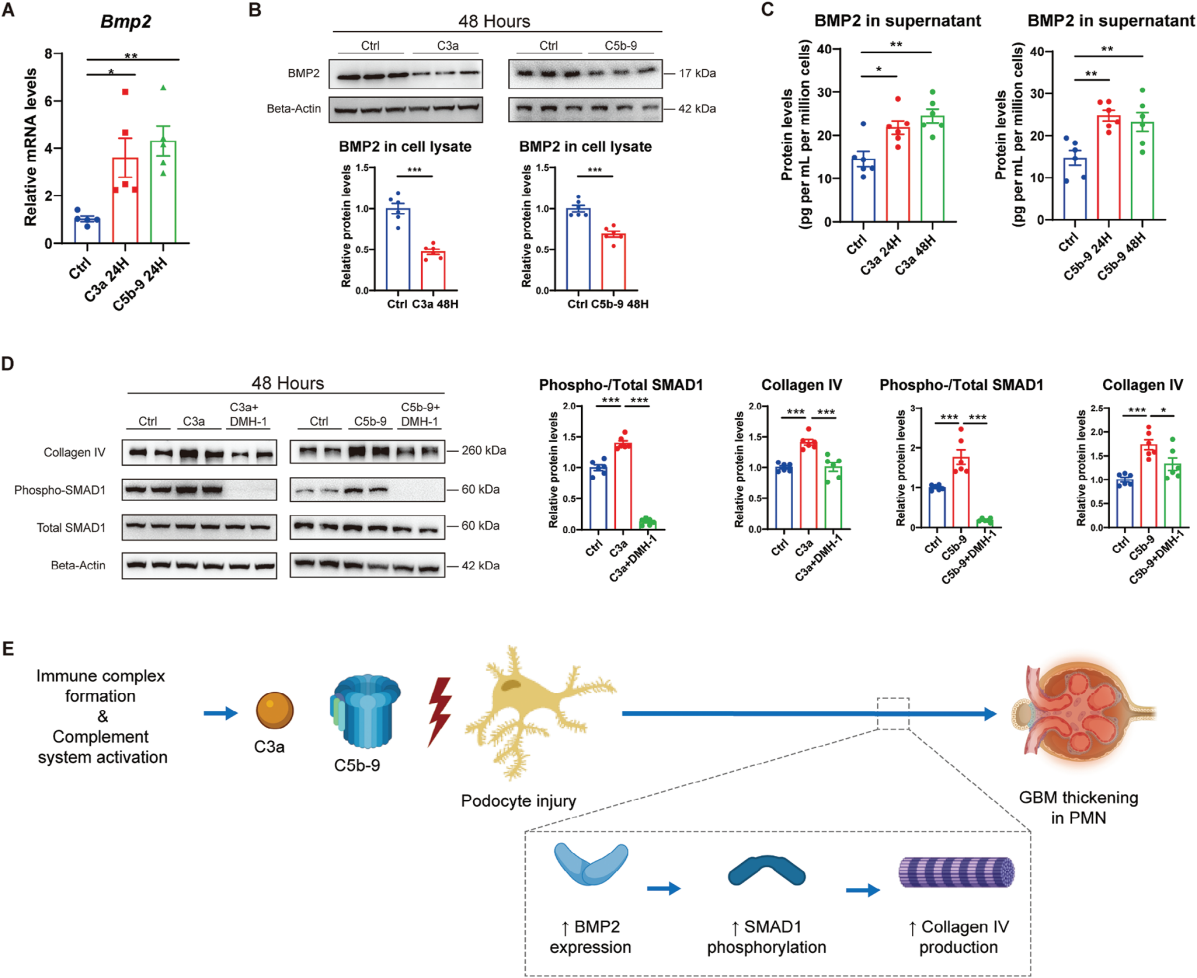
Complement‐induced podocyte BMP2/pSMAD1/COL4 signaling activation in vitro. A) qPCR analysis showing podocyte mRNA levels of *Bmp2* after treatment with C3a and C5b‐9 for 24 h. B) Western blot analysis showing the protein levels of BMP2 in podocyte cell lysates after treatment with C3a and C5b‐9 for 48 h. Beta‐actin was used as the loading control. C) Enzyme‐linked immunosorbent assay showing the protein levels of BMP2 in podocyte cell culture supernatant after treatment with C3a and C5b‐9 for different durations. D) Western blot analysis showing the protein levels of collagen IV and phosphorylated and total SMAD1 in podocyte cell lysates after incubation with C3a, C3a+DMH‐1, C5b‐9, and C5b‐9+DMH‐1 for 48 h, with the corresponding quantification. Beta‐actin was used as the loading control. E) Schematic hypothesis of complement‐induced BMP2/pSMAD1/COL4 pathway activation of podocytes in PMN. *P < 0.05; **P < 0.01; ***P < 0.001; NS, not significant. Each experiment was replicated for 5 times for A‐D. N = 5 per group for A, and N = 6 per group for B–D. Data are presented as mean ± SEM. One‐way ANOVA test for A, C, and D; and Student's two‐tailed t‐test for B.

Next, we investigated the culprit that might cause BMP2 upregulation in PMN. Complement deposits are common and considered the key to podocyte injury in PMN.^[^ ^3^, ^16^ ^]^ For this reason, we tested complement C3a and C5b‐9, two components abundantly depositing in PMN and initiating maladaptive pathways in podocytes.^[^ ^3^, ^17^ ^]^ Incubation with C3a or C5b‐9 for 24 h increased *Bmp2* mRNA levels in mouse podocytes (**Figure** **4**A). Surprisingly, we detected reduced BMP2 protein levels in podocyte lysates at 48 h post‐treatment (Figure 4B). As we observed, the 17‐kDa BMP2 protein band represents the glycosylated, soluble form.^[^ ^18^ ^]^ Considering the increased *BMP2* mRNA levels after treatment, a possible explanation could be the secretion of synthesized BMP2. Hence, we measured BMP2 protein levels in the cell culture medium supernatant and found that consistent with the qPCR results, complement C3a and C5b‐9 were all able to induce BMP2 secretion from podocytes (Figure 4C). Subsequently, we tested the downstream pathway, i.e., SMAD1 phosphorylation and collagen IV expression. Stimulation with C3a and C5b‐9 augmented SMAD1 phosphorylation and increased collagen IV expression at 48 h post‐stimulation (Figure 4D). Furthermore, inhibition of BMP2 through its specific inhibitor DMH‐1^[^ ^19^ ^]^ blocked SMAD1 phosphorylation and abolished C3a‐ and C5b‐9‐induced collagen IV augmentation at 48 h (Figure 4D). Our investigations on podocytes bridge two principal pathological changes of PMN, i.e., immune complex depositing and GBM thickening (Figure 4E).

### 2.4 Activation of M2‐like Macrophages in PMN

**Figure 5:**
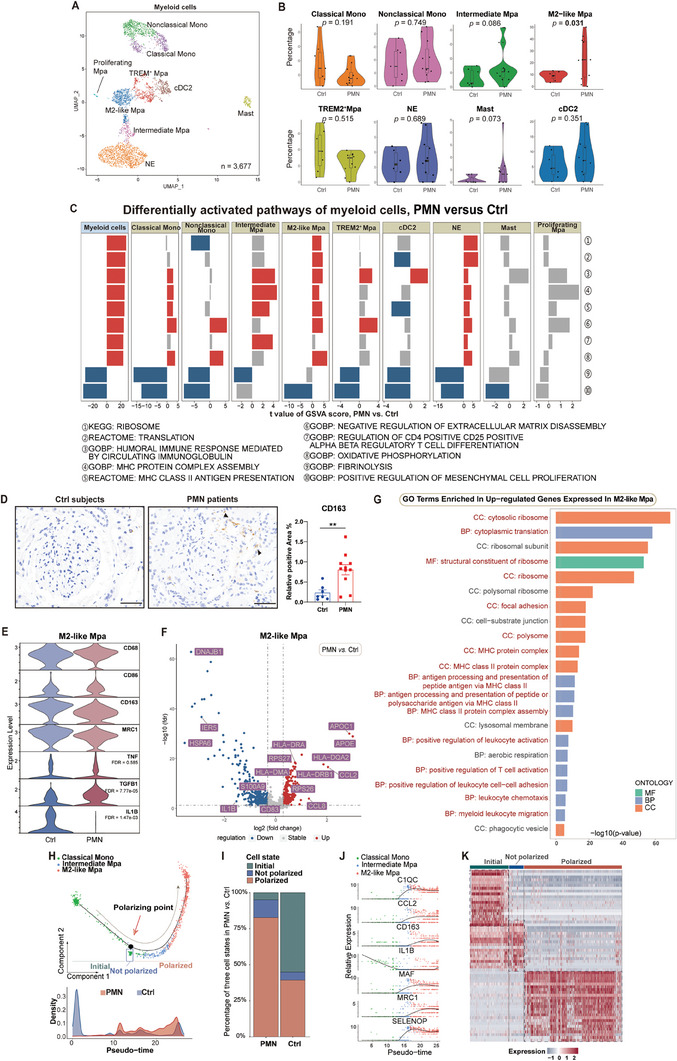
Activation of M2‐like macrophages in PMN. A) UMAP indicating subclustered myeloid leukocytes color‐coded by different cell types. B) Respective cell percentages of myeloid leukocytes in PMN versus Ctrl. C) Major upregulated (red) and downregulated (blue) pathways of myeloid cells in PMN versus Ctrl, according to GSVA. D) Immunohistochemistry analysis of CD163 expression in paraffin‐embedded kidney biopsy sections of PMN patients (left) and control subjects (right), with corresponding quantification. Black arrowheads indicate CD163‐positive cell infiltration. E) Violin plot showing the expression levels of macrophage M1 and M2 polarization marker genes in M2‐like Mpa. F) Volcano plot showing major upregulated (red) and downregulated (blue) genes in M2‐like macrophages. G) GO enrichment presenting major upregulated pathways of M2‐like Mpa, with pathways of interest marked red. H) Pseudotime trajectory analysis presenting the polarization trajectory from classical monocytes to intermediate macrophages and finally M2‐like macrophages (upper) and their distribution from PMN and Ctrl along the inferred differentiation process (lower). I) Respective cell percentages of H in PMN versus Ctrl. J) Expression of marker and functional genes along the polarization trajectory from classical monocytes to intermediate Mpa and finally to M2‐like Mpa in PMN. K) Heatmap indicating distinct gene expression patterns of different cell types in H. **P < 0.01. Scale bar = 50 µm. N = 7 for Ctrl, N = 11 for PMN for D. Data are presented as mean ± SEM. Student's two‐tailed t‐test for D. See also Figure S6 (Supporting Information).

PMN is considered a renal‐limited autoimmune disease, therefore, the transcriptomic signatures of renal‐infiltrated immune cells should be valuable resources to reveal the mystery of PMN pathophysiology. Myeloid leukocytes are indispensable in autoimmune diseases due to their roles in antigen processing and presentation, regulation of inflammatory responses, and secretion of cytokines.^[^ ^20^ ^]^ Here, we subclustered myeloid cells (N = 3677) as M2‐like macrophages, intermediate macrophages without apparent M1 or M2 polarization, *TREM2^+^* lipid‐associated macrophages, proliferating macrophages, *CD14^+^* classical monocytes, *CD16^+^* nonclassical monocytes, type 2 conventional dendritic cells (cDC2), NEs, and mast cells (**Figure** **5**A; Figure S6A–D, Supporting Information). A markedly higher percentage of M2‐like macrophages was observed in PMN (Figure 5B; Tables S2–S4, Supporting Information), confirmed by immunohistochemistry staining of the M2 macrophage marker CD163 (Figure 5D). Differentially activated pathways implied enhanced gene translation, antigen presentation via MHC class II, CD4^+^ T‐cell activation, and aerobic respiration of myeloid cells in PMN, which showed consistency in most cell subtypes except nonclassical monocytes and cDC2, and the differences are not significant in mast cells and proliferating macrophages; on the contrary, their functions of promoting mesenchymal cell proliferation and ECM degradation were inhibited (Figure 5C).

We then studied M2‐like macrophages (N = 540) for their increased infiltration in PMN. In the PMN kidneys, M2‐like macrophages displayed a complex phenotype and expressed the markers *CD86*, *CD163*, and *MRC1*, associated with wound‐healing M2a, immunoregulating M2b, and immunosuppressive M2c subsets, respectively (Figure 5E; Figure S6E, Supporting Information). Their complicated functions were specified by comparing DEGs in PMN versus Ctrls: first, PMN samples showed increased expression levels of *APOC1* and *APOE* in response to dyslipidemia in PMN (Figure 5F,G). Moreover, as key antigen‐presenting cells (APCs), these macrophages presented high levels of MHC class II, such as *HLA‐DQA2*, *HLA‐DRA*, *HLA‐DRB1*, *HLA‐DPA1*, and *HLA‐DQA1*, indicating enhanced exogenous antigen processing and presentation to activate leukocytes (Figure 5F,G). In addition, two potent chemokines of monocyte recruitment, i.e., *CCL2* and *CCL8*, were highly expressed in these macrophages (Figure 5F,G). Downregulated genes included *S100A9*, which promotes M1 polarization, and inflammation mediator *IL1B*, suggesting an anti‐inflammatory profile of these macrophages (Figure 5F). Furthermore, several HSP genes, such as *DNAJB1* and *HSPA6*, showed apparent downregulation (Figure 5F). Similar activation patterns of other myeloid cells in PMN were found by GO enrichment, with classical and nonclassical monocytes appearing to activate B cells and *TREM2^+^* lipid‐associated macrophages presenting active endocytosis and response to lipoprotein particles (Figure S6G, Supporting Information).

The polarization of mononuclear phagocytes presented a continuous trajectory from classical monocytes to intermediate macrophages and finally to M2‐like macrophages, with a clear point where a group of nonpolarized monocytes had branched off (Figure 5H). During the polarization process in PMN patients, consistent with the changes in cell composition in myeloid leukocytes, fewer cells were in the initial state (4.90% in PMN versus 55.04% in Ctrls), while vastly more cells were M2‐polarized (82.76% in PMN vs 39.29% in Ctrls), implying active M2 polarization in PMN (Figure 5H,I). Additionally, cells of different states presented distinct transcriptomic features (Figure 5J,K): following polarization, downregulation of the inflammatory cytokine *IL1B* and upregulation of *C1qC*, *CCL2*, *MAF*, *CD163*, *MRC1*, and *SELENOP* were observed in PMN (Figure 5J; Figure S6F, Supporting Information). Similar patterns were found in Ctrls, but *CCL2* remained silent, and the expression levels of other functional genes differed between PMN and Ctrl (Figure S6F, Supporting Information). Collectively, these results reveal the origin, polarization, and anti‐inflammatory phenotype of macrophages in PMN.

### 2.5 Changes in Memory B and Plasma Cells in PMN

**Figure 6:**
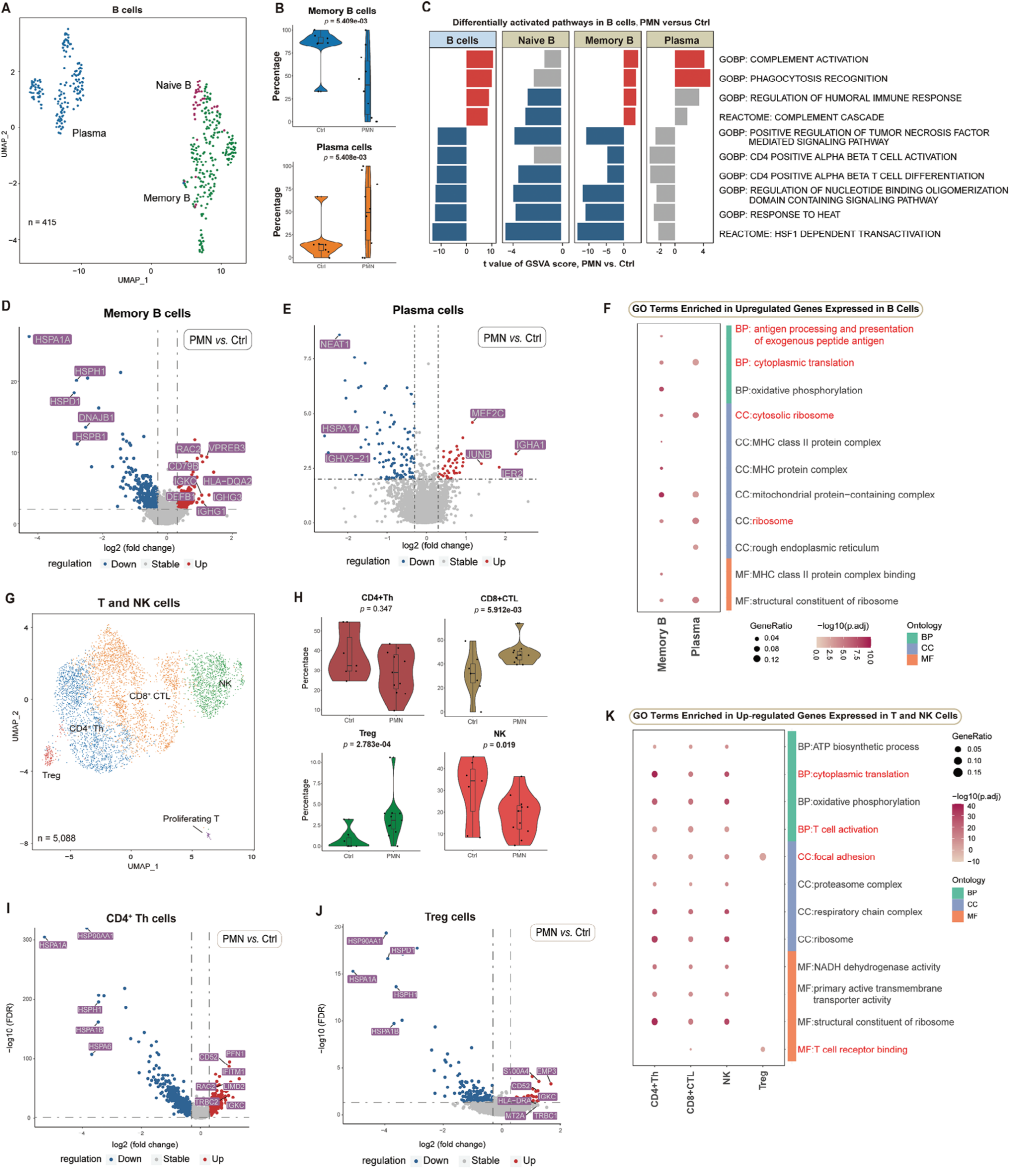
Transcriptomic signatures of B and T lymphocytes in PMN. A) UMAP indicating subclustered B lymphocytes color‐coded by different cell types. B) Respective cell percentages of B lymphocytes in PMN versus Ctrl. C) Major upregulated (red) and downregulated (blue) pathways of B lymphocytes in PMN versus Ctrl, according to GSVA. D) Volcano plot showing major upregulated (red) and downregulated (blue) genes in memory B cells. E) Volcano plot showing major upregulated (red) and downregulated (blue) genes in plasma cells. F) GO analysis presenting major upregulated pathways of memory B cells and plasma cells, with pathways of interest marked red. G) UMAP indicating subclustered T and NK cells color‐coded by different cell types. H) Respective cell percentages of T and NK cells in PMN versus Ctrl. I) Volcano plot showing major upregulated (red) and downregulated (blue) genes in *CD4^+^* Th cells. J) Volcano plot showing major upregulated (red) and downregulated (blue) genes in *Foxp3^+^* Treg cells. K) GO enrichment presenting major upregulated pathways of T and NK cells. See also Figures S7 and S8 (Supporting Information).

Another key to decoding the etiology of PMN lies in B lymphocytes, which serve as both APCs and the producer of antibodies.^[^ ^3a^ ^]^ B cells (N = 415) were subclustered as naïve B cells, memory B cells, and plasma cells (**Figure** **6**A; Figure S7A–D, Supporting Information). PMN samples showed increased plasma‐cell infiltration accompanied by a lower percentage of memory B cells, suggesting B‐cell maturation and activation (Figure 6B; Tables S2–S4, Supporting Information). Additionally, pathways such as complement activation, phagocytosis, and humoral immune response were activated in memory B and plasma cells, but not in naïve B cells; while the HSPs, NOD pathway, CD4^+^ T‐cell activation, and TNF signaling were downregulated in all B lymphocytes in PMN (Figure 6C).

Next, we focused on infiltrated memory B cells (N = 218) and plasma cells (N = 163). For functional analysis, volcano plots and GO terms exhibited augmented antigen processing and presentation by *HLA‐DQA2* and ribosome activity of memory B cells (Figure 6D,F). Specifically, the volcano plot highlighted the upregulation of *IER2*, *JUNB*, and *MEF2C* in plasma cells (Figure 6E). Although the significance of *IER2* upregulation remains unclear, augmented *JUNB* and *MEF2C* might participate in B‐cell development and maturation.^[^ ^21^ ^]^ Similarly, GO terms also suggested active cytoplasmic translation in plasma cells (Figure 6F), implying enhanced antibody production. Therefore, we further explored *IGHG* expression levels in memory B and plasma cells. Regarding the overall DEG results, *IGHG1*, *IGHG2*, and *IGHG3* were upregulated in PMN memory B cells, while *IGHGs* in plasma cells seemed not to present significant differences (Figure S7E, Supporting Information). Nevertheless, in the split violin plot (Figure S7F, Supporting Information), more PMN individuals expressed *IGHGs* in both memory B and plasma cells, indicating active antibody synthesis in PMN patients. According to trajectory analysis, during the differentiation process from memory B to plasma cells, the HLA complex and the immune activation molecules *CD83* and *CD69* were downregulated. In contrast, these cells presented upregulated ribosomal activity, immunoglobulin synthesis, and cellular respiration (Figure S7G, Supporting Information). In total, we reported augmented infiltration of plasma cells and activation of local renal B cells in PMN.

### 2.6 Immunosuppressive Trends of T Lymphocytes in PMN

T lymphocytes are also critical regulators of the immune system and autoimmune diseases.^[^ ^22^ ^]^ We subclustered T and NK cells (N = 5088) in our samples as *CD4^+^* Th cells, *CD8^+^* CTLs, *Foxp3^+^* Treg cells, NK cells, and proliferating T cells (Figure 6G; Figure S8A–D, Supporting Information).

In contrast to previous studies, which reported decreased Treg percentages in the peripheral blood of PMN patients,^[^ ^22^, ^23^ ^]^ our data suggested increased Treg cells in PMN kidneys (Figure 6H; Tables S2–S4, Supporting Information). Upon analyzing their DEGs, Th cells and Treg cells displayed very limited numbers of and low fold changes in upregulated genes and appeared to be immunosuppressive (Figure 6I and J): we found elevated *CD52* expression levels in both Th cells and Treg cells, an immune suppressor downregulated in systemic lupus erythematosus.^[^ ^24^ ^]^ Additionally, *EMP3* was augmented in Treg cells, which was associated with immunosuppression and T‐cell exclusion in glioblastoma.^[^ ^25^ ^]^ Furthermore, these cells showed apparent downregulation of various HSPs, including *HSPA1A* and *HSP90AA1* (Figure 6I and J). GO enrichment analysis implied increased activation of Th cells and T‐cell receptor binding activity of Treg cells, with active aerobic respiration and ribosome activities in T and NK cells (Figure 6K). We thus concluded that Th cells and Treg cells in PMN might play an immunosuppressive role, whereas the underlying driving force deserves further exploration. Moreover, although we observed more CTLs in the diseased kidneys (Figure 6H; Tables S2–S4, Supporting Information), they showed very limited upregulated genes and unspecific downregulated genes. Their upregulated pathways mainly involved ribosome activity and respiratory chain, while downregulated genes were majorly enriched in protein folding chaperone pathways (Figure S8F, Supporting Information).

### 2.7 Intercellular Crosstalk in PMN

**Figure 7:**
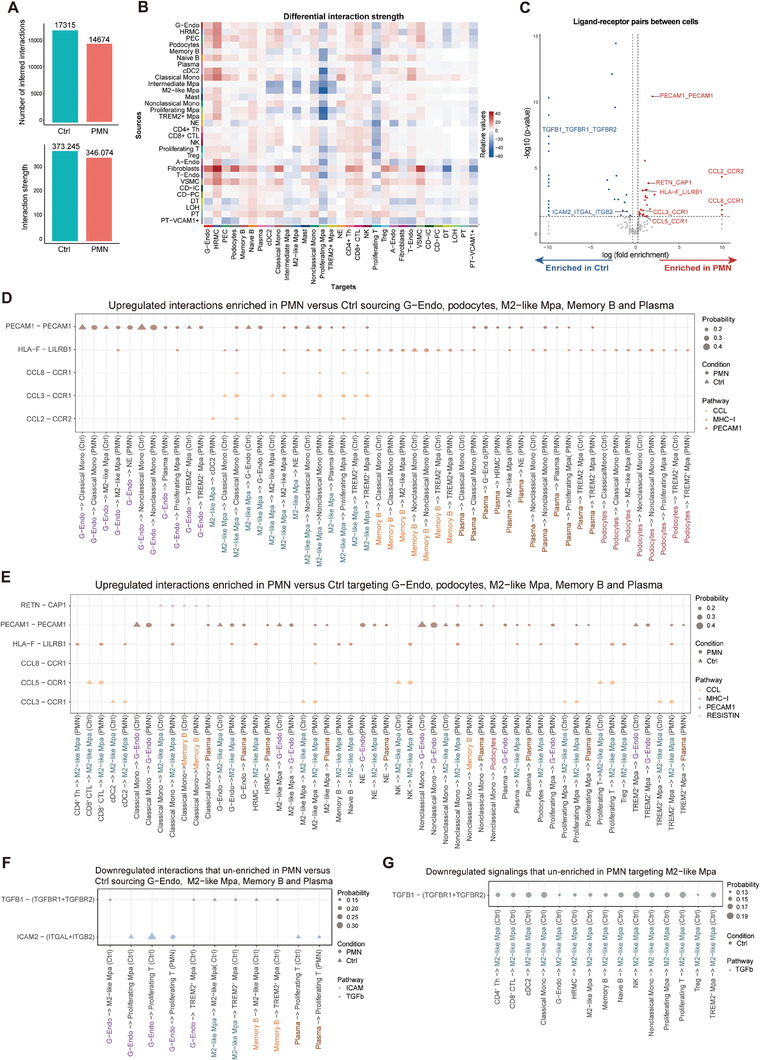
Intercellular crosstalk in PMN. A) Bar plots showing overall numbers (above) and strength (below) of inferred interactions among cell types in PMN versus Ctrl. B) Heatmap showing differential interaction strength sourcing and targeting different cell types in PMN versus Ctrl. C) Volcano plot showing enrichment of significant interactions among cell types within PMN versus Ctrl. Enrichment of interactions is plotted as significance versus fold enrichment. The labeled points are signals that are either significantly upregulated and enriched in PMN (red) or significantly downregulated and un‐enriched in PMN (blue) relative to Ctrl. D) Dot plots showing major upregulated interactions enriched in PMN versus Ctrl sourcing G−Endo (purple), podocytes (red), M2−like Mpa (green), memory B (orange), and plasmas (brown). E) Dot plots showing major upregulated interactions enriched in PMN versus Ctrl targeting G−Endo (purple), podocytes (red), M2−like Mpa (green), memory B (orange), and plasmas (brown). F) Dot plots showing interactions downregulated and un‐enriched in PMN, sourcing G−Endo (purple), M2−like Mpa (green), memory B (orange), and plasmas (brown). G) Dot plots showing the interaction downregulated and un‐enriched in PMN, targeting M2−like Mpa (green). In D‐G, dot sizes indicate the probabilities of interactions active between two cell types, dot colors indicate the pathways that an interaction belongs to, and PMN and Ctrl were respectively indicated by round dots and triangle dots.

No cell is an island. Cells communicate and interact with each other to play their roles. Hence, we analyzed the ligand‐receptor pairs to provide information on cell‐cell crosstalk in PMN versus Ctrl. Overall, both the total number and total strength of cellular interactions were slightly reduced in PMN (**Figure** **7**A). We focused on several ligand‐receptor pairs with significantly differential expression between PMN and Ctrl, and how they changed in five cell types closely related to PMN (Podo, G‐Endo, M2‐like Mpa, Memory B, and Plasma cells) (Figure 7B,C). In detail, we noticed increased PECAM1‐PECAM1 interaction, where one side is plasma cells and M2‐like macrophages, and the other side G‐Endo, HRMC, and multiple mononuclear phagocytes (Figure 7D,E), suggesting augmented immune cell adhesion and activation.^[^ ^26^ ^]^ Another upregulated cellular crosstalk was HLA‐F sourced podocytes, memory B cells, plasma cells, and M2‐like macrophages to target the inhibitory receptor LIL1RB on M2‐like macrophages (Figure 7D,E), which tended to be protective against phagocytosis.^[^ ^27^ ^]^ Moreover, M2‐like macrophages communicated with various mononuclear phagocytes via producing chemokines CCL2, CCL3, and CCL8 to bind receptor CCR1 and CCR2 (Figure 7D); as targets, they also received chemoattractant CCL3 and CCL8 from mononuclear phagocytes, and CCL5 from T cells and NK cells (Figure 7E). We also found the RETN‐ CAP1 interaction from classical and non‐classical monocytes targeting podocytes, memory B cells, plasma cells, and M2‐like macrophages (Figure 7E). Such an interaction was reported to be associated with impaired mitochondrial homeostasis in metabolic diseases,^[^ ^28^ ^]^ and macrophage proliferation and osteoclastic activities in osteoporosis,^[^ ^29^ ^]^ whereas its possible role in PMN kidney needs further investigations. Downregulated intercellular communication involved plasma cell‐sourced ICAM2 targeting integrins on proliferating T cells, and TGFB1 from various immune cells and glomerular cells to bind TGFBR1 and TGFBR2 on M2‐like macrophages (Figure 7F,G). Decreased ICAM2‐integrin signal implied reduced leukocyte adhesion and interaction,^[^ ^30^ ^]^ while downregulated TGFB1‐TGFBR crosstalk disapproved the possibility of macrophage as a source of ECM in the PMN kidney.^[^ ^31^ ^]^ In short, cell‐cell crosstalk highlighted the importance of the delicate immune network in the PMN kidney.

## 3 Discussion

As the most common cause of nephrotic syndrome in non‐diabetic adults and a leading cause of ESRD, PMN received increasing attention due to its rising incidence (increased by 13% annually in China) associated with air pollution.^[^ ^3^, ^32^ ^]^ Single‐cell methodology to decode the pathophysiology of PMN is promising, whereas its application in nephrology is hampered by the few cells obtained once in a core needle biopsy.^[^ ^6^ ^]^ Owing to the lack of enough podocytes detected, a previous scRNA‐seq study of anti‐PLA2R PMN failed to explore podocyte‐specific alterations, which limited their translational significance.^[^ ^33^ ^]^ To overcome such a problem, we scaled up our sampling to involve eleven PMN patients and seven healthy subjects, and acquired an adequate number of podocytes (together with other renal inherent and immune cells) to identify pathogenic pathways in the disease, ensuring the innovation and significance of our study.

PMN is considered a renal‐limited, autoantibody‐induced, complement‐mediated, podocyte‐targeted autoimmune disease.^[^ ^3^, ^4^, ^5^, ^16^ ^]^ The histopathological characteristic of PMN is GBM thickening and the formation of subepithelial “spikes” due to ECM accumulation.^[^ ^4^ ^]^ COL4A3 and COL4A4 are two of the major ECM components of GBM,^[^ ^11^ ^]^ observed to be upregulated in both human and experimental MN,^[^ ^34^ ^]^ but their upstream regulator remains largely unclear. Our data demonstrated elevated BMP2 expression in PMN podocytes, which could augment the expression of COL4A3 and COL4A4 in podocytes. To detect the activation of the BMP2 signaling pathway, we measured its canonical intracellular messenger SMAD1^[^ ^10^ ^]^ and found that SMAD1 phosphorylation coordinates with upstream BMP2 stimulation and downstream collagen IV production, thereby identifying the BMP2/pSMAD1/COL4 signaling pathway in the pathogenesis of PMN. Electron microscopy of PMN is characterized by subepithelial electron‐dense deposits,^[^ ^4^ ^]^ referring to immune complexes that activate the complement system to induce podocyte injury and lead to collagen IV isoform expression in PMN.^[^ ^3^, ^35^ ^]^ Therefore, we hypothesized and then proved that BMP2 elevation could be triggered by complement components to mediate the BMP2/pSMAD1/COL4 pathway in podocytes. C3a and C5b‐9 represent common products of the complement activating pathways,^[^ ^36^ ^]^ suggesting that the BMP2/pSMAD1/COL4 pathway should be a common phenomenon that links the pathomechanisms of complement system activation and GBM thickening in PMN. Moreover, the specific signal transduction process between complement stimulation and BMP2 upregulation deserves further exploration, and cAMP might be a potential second messenger, as C3a and C5b‐9 can elicit a decrease in intracellular cAMP.^[^ ^17^, ^37^ ^]^ Another finding is that podocytes showed augmented expression of class I HLA complex, which presents endogenous peptides to CD8^+^ T cells^[^ ^38^ ^]^ and NK cells.^[^ ^39^ ^]^ However, in our data, CTLs and NKs only presented very limited numbers of and low fold changes in upregulated genes and unspecific alterations in downregulated genes, which could barely be associated with the specific pathogenic mechanism of PMN, and were consistent with our current knowledge that CTLs would not play a major role in PMN. Further research should explore the association between the podocyte expression of class I HLA molecules and PMN vulnerability and pathogenesis.

Immune cells stand for another crucial perspective in PMN. APCs, primarily mononuclear phagocytes, play a pivotal role in the onset of anti‐PLA2R autoantibody production, involving loss of tolerance to circulating soluble PLA2R and activation of alveolar macrophages.^[^ ^16^, ^32^, ^40^ ^]^ In the PMN kidney, we found an elevated percentage of M2‐like macrophages with complicated functions, mainly anti‐inflammatory. M2 macrophages include heterogeneous subpopulations with different phenotypes and functions. M2a macrophages marked by CD206 and M2b macrophages marked by CD86 may be involved in PMN by promoting the deposition of IgG subtypes and activating complements, while M2c macrophages marked by CD163 may be protective in PMN by reducing the production of C4d.^[^ ^41^ ^]^ In our study, we found M2 macrophages expressed markers of M2a, M2b, and M2c, suggesting that these cells should promote IgG production and regulate the complement system in the pathogenesis of PMN. Moreover, elevated expression of apolipoproteins, HLA class II, and chemokines were detected in these M2 macrophages. Therefore, they should also participate in PMN pathogenesis by regulating lipid metabolism, presenting antigens, and attracting other immune cells. Similarly, previous studies have reported M2‐dominant macrophage infiltration in lupus nephritis, IgA nephropathy, and PMN,^[^ ^41^, ^42^ ^]^ which implied that M2 polarization might be associated with complement activation.^[^ ^41^, ^42^ ^]^ However, considering the complexity of various complement components that are deposited in PMN,^[^ ^17a^ ^]^ the associations between specific complement components and macrophages in PMN need further exploration.

Local B lymphocytes displayed overall activated features: memory B cells presented robust antigen processing and presentation functions, and were prone to producing more IgG antibodies in PMN; plasma cells showed augmented renal infiltration with increased expression of IgG. Our results provide new insights into anti‐B‐cell therapy in PMN:^[^ ^43^ ^]^ patients might benefit from its effect on not only circulating but renal B cells, and therapies to protect against plasma cells would be a potential target.

Regarding T lymphocytes, former research described reduced Treg percentages in the peripheral blood of PMN patients.^[^ ^22^, ^23^ ^]^ Nevertheless, Th cells and Treg cells in our PMN samples displayed very limited activation, with elevated expression levels of several immunosuppressive genes. Another finding is that the percentage of infiltrated CTLs increased in PMN, but they only presented very limited upregulated genes and unspecific downregulated genes, which could barely be associated with the specific pathogenic mechanism of PMN, and were consistent with our current knowledge that CTLs would not play a major role in PMN.

Renal tubular cells, likely to be a bystander of PMN pathogenesis, presented no significant changes that could be associated with PMN pathophysiology, and related information was discussed in Supporting Results.

There are several limitations of our current study. First, our data were based on kidney biopsy samples, which limited our access to circulating immune cells, especially the producers of anti‐PLA2R autoantibodies.^[^ ^44^ ^]^ Future studies could collect both kidney biopsies and peripheral blood samples from the same patients for a more robust analysis. Besides, our sequencing data were based on the Asian population, which could impact the results of several analyses, such as the HLA molecules expressed on immune cells. The inclusion of subjects from other populations would extend our findings. In addition, we did not confirm our findings in vivo using rodent models, as mice lack PLA2R expression on podocytes. The recently developed transgenic mouse line expressing PLA2R1 on podocytes would help to address such a problem, and contribute to the development of intervention methods toward PMN.^[^ ^45^ ^]^ Moreover, we collected a certain number of podocytes allowing us to perform analysis and reach a significant threshold, but the number was still relatively small. Therefore, we appeal to investigators to collect more PMN biopsies for scRNA‐seq and perform analysis in combination with our dataset. Additionally, transcriptomic analysis at the single‐cell level helps to unravel the cell‐specific gene expression patterns, whereas some spatial information could be discarded during processing. Besides, validations at the protein level are limited due to the methodology of Western blot and immunostaining. Regarding these limitations, multi‐omics such as single‐cell sequencing combined with spatial transcriptomics and proteomics should benefit future biomedical investigations.

In summary, we provide, to our knowledge, the largest scRNA‐seq dataset of anti‐PLA2R autoantibody‐positive PMN. We prepared a comprehensive transcriptomic profile of the kidney in PMN and reported substantial alterations in the diseased kidney, including podocytes, renal‐infiltrated mononuclear phagocytes, and B cells. Our diverse bioinformatic, in vivo, and in vitro approaches will serve as a paradigm for other investigations of glomerular diseases. Our valuable data contribute to the complete understanding of PMN pathophysiology, and provide new insights into its therapeutic targets.

## 4 Experimental Section

### Patients

All patient samples were acquired from the nephrology department of Renji Hospital, and taken by kidney biopsy to confirm their cause of proteinuria. Patient inclusion criteria include: newly diagnosed PMN patients with positive anti‐PLA2R autoantibody in serum, histopathological diagnosis of MN stage I‐II, and without other kidney diseases. Control subjects were healthy kidney transplantation donors from the urology department of Renji Hospital, and all control samples were confirmed to have no pathological alterations via histopathological analysis. Details were shown in Table 1.

### Tissue Processing and Single‐Cell Dissociation

The renal biopsies were preserved in cold MACS Tissue Storage Solution (Miltenyi Biotec), minced into small pieces with a razor blade, and incubated at 37 °C in freshly prepared dissociation buffer containing enzymes from a Multi Tissue Dissociation Kit (Miltenyi Biotec) with a rotation speed of 200 rpm. Dissociated cells were harvested every 10 min by filtering the cell suspension through a 70‐µm cell strainer (FALCON) into 10% FBS buffer on ice. The residual biopsy tissue was dissociated again with 1 ml dissociation buffer for 10 min and passed through the cell strainer into the same FBS buffer from the first collection. This dissociation procedure was repeated three times until most of the tissue had been separated into single cells (total dissociation time was 30 min). Finally, the cells were collected by centrifugation at 1500 rpm for 5 min, resuspended in PBS containing 5% FBS, and strained through a 40‐µm cell strainer (FALCON) to further remove cell clumps and large fragments. Cell viability was approximately 90% for the biopsy used in this study, as assessed by Trypan Blue staining.

### Single‐Cell RNA Sequencing and Library Construction

Library construction was performed using the Chromium Single Cell 3′ Library & Gel Bead Kit v3.1 (10X Genomics) according to the manufacturer's instructions. Briefly, cells were mixed with reverse transcriptase into Gel Beads‐In‐Emulsions (GEMs). mRNA was released after cell lysis inside the droplets, captured by poly‐dT sequences, and became cDNA by RT‐PCR, which was used for library preparation. Libraries were run on NovaSeq 6000 PE150 sequencing by APExBIO Technology LLC. (Shanghai China).

### Single‐Cell RNA‐Seq Data Processing

The Cell Ranger toolkit (v3.0.2) provided by 10X Genomics was used to demultiplex and align the transcriptome data of each sample to the human reference genome (GRCH38) to generate the gene‐cell unique molecular identifiers (UMIs). For each sample, cells expressing less than 200 genes or more than 6000 genes, cells with proportions of mitochondrial genes that were more than 20%, and cells contaminated by erythroid markers (HBB1 and HBE1) were discarded. The package DoubletFinder^[^ ^46^ ^]^ based on R and the package Scrublet^[^ ^47^ ^]^ based on Python were applied to doublet detection; cells determined to be doublets by both methods were excluded from the data analysis. We filtered all the mitochondrial genes and genes expressed in less than 3 cells (default). Thus, 72 374 high‐quality cells and 27 387 genes were retained for subsequent analysis using the R package Seurat (v4.1.0).^[^ ^48^ ^]^ The transcriptome data matrix was log‐transformed with a scale factor of 10 000 (default).

### Single‐Cell Data Clustering and Sub‐Clustering

For clustering all 72374 cells from 18 samples, canonical correlation analysis (CCA),^[^ ^49^ ^]^ a built‐in method for data integration in the package Seurat, was utilized to remove batch effects from 18 samples, in which each sample was assigned to one batch. A total of 2000 genes with the highest variance were selected to identify anchors between 18 samples, and the anchors were integrated into one Seurat object. Then, the Seurat package was used to perform linear (principal component analysis (PCA)^[^ ^50^ ^]^) and nonlinear (uniform manifold approximation and projection (UMAP)^[^ ^51^ ^]^) dimensional reduction and clustering on these integrated data using the aforementioned 2000 highly variable genes. The first 40 principal components (PCs) were used for performing UMAP. The resolution parameter in the “FindClusters” function was set as 1.1 for clustering, and the Wilcoxon rank‐sum test (default) performed by the function “FindAllMarkers” was used to find differentially expressed genes of each cluster as its signatures (setting: min.pct = 0.25, logfc.threshold = 0.25). Then, the clusters were refined and annotated according to the specific expression pattern of the canonical cell type markers or detected cluster markers. Finally, the quality and rationality of clustering were checked by heatmap plots and dot plots that profiled the expression level of marker genes in each cell cluster we defined.

For sub‐clustering, PCA was performed based on the 2000 most variable genes in the scRNA‐seq profile of each cell category, the “RunHarmony” function within the R package Harmony^[^ ^52^ ^]^ was performed after PCA was used to remove batch effects, and Harmony embeddings were used for further dimensional reduction and visualization by UMAP. Parameters were adjusted for each cell category, including the number of significant dimensions of Harmony embeddings and clustering resolution. Likewise, cell markers proven by the literature or experiments and cluster signatures identified by the Wilcoxon rank‐sum test were used to define the cell types, and heatmap plots were used to check the results of clustering.

Note that we found abnormally increased counts of B cells from MN11 caused by infection rather than PMN, and these cells were excluded from the subclustering of the B cell category to avoid any biases or batch effects.

### Test of Changes in Cell Components Between PMN and Control

For the anatomy of kidneys and sampling locations of renal biopsies varies in individuals, percentage changes between PMN and control cells were tested and depicted for each cell subtype out of glomerulus, renal interstitial cells, B cells, myeloid immunocytes, T cells, and tubules, respectively. The significance of differential cell components was tested utilizing a differential abundance test based on generalized linear mixed models (GLMM) methodology, which fits GLMMs for each cluster with cell counts as binomial response variables, conditions (PMN or Control) as fixed effects and individuals as random effects, respectively, and then calculates differential tests separately for each cell subtypes.^[^ ^53^ ^]^ The calculation was implemented by R package lm4^[^ ^54^ ^]^ and multcomp.^[^ ^55^ ^]^

Note that it was found abnormally increased counts of B cells from MN11 as well as NE from Ctrl6 caused by infection rather than PMN, and these cells were excluded from the differential analysis on cell components to avoid any biases. Cell subtypes with less than 5 cells in at least 5 samples were also filtered out of the test.

### Analysis of Differentially Activated Pathways Based on GSVA

In order to investigate how the functions of cells in the glomerulus, B cell(except B cells from MN11), and myeloid cell categories change in PMN, gene set variation analysis (GSVA)^[^ ^9^ ^]^ with “ssgsea” method^[^ ^56^ ^]^ was used to calculate GSVA scores to evaluate the activity of certain pathways for each cell. Pathways defined by Gene Ontology (GO, http://geneontology.org), Kyoto Encyclopedia of Genes and Genomes (KEGG, https://www.genome.jp/kegg), and Reactome (https://reactome.org) were included in GSVA. To identify the differentially activated pathways, a differential analysis of GSVA scores of all those pathways in PMN and Ctrl cells was performed by Limma.^[^ ^57^ ^]^ Positive t values indicate that the pathways are activated in PMN cells, while negative t values indicate the inhibition of the pathways in PMN cells. Higher absolute t values indicate more significant results.

### Identification of Differentially Expressed Genes between PMN and Ctrl

Differentially expressed genes (DEGs) between PMN and Ctrl in various cell types were identified using the function “FindMarkers” in Seurat (v4.1.0) with the “MAST”^[^ ^58^ ^]^ method, which is tailored to the scRNA‐seq transcriptome. Genes expressed in more than 10% of cells in either of two conditions were considered in the analysis, and the UMI counts were used as inputs. Note that B cells from MN11 were not included in the differential expression analysis for B cell categories. False discovery rates (FDR) were used to adjust the *P*‐values of the tests to reduce false positives.

In order to figure out whether the differential expressed genes are common in the majority of PMN patients, it was further identified unbiased DEGs based on random sub‐sampling for cell subtypes in which most cells were from one or two individuals. Specifically, for cell subcluster *i*, the outlier of cell counts was defined as

(1) $$Outlier_{i}=\;Q3_{i}+1.5\times \left(Q3_{i}-Q1_{i}\right)$$

in which *Q*1_*i*_ and *Q*3_*i*_are the first and the third quartiles of cell counts of the cell type *i*, respectively, and *Q*3 − *Q*1 was known as the interquartile range (IQR). If a subcluster had any subjects with cell counts of more than *Outlier_i_*, for this cell cluster, it was found out the subjects with cell counts exceeding *Outlier_i_*, from which cells were randomly sub‐sampled to keep the number of cells at an average level of the corresponding cell cluster. These sub‐sampled cells were then used to represent all cells of the subjects to perform DEGs identification for this cell cluster. This random sub‐sampling process was implemented for 100 times to detect common DEGs. Theoretically, these DEGs identified based on random sub‐sampling were scarcely biased by certain subjects with dominant numbers of cells. Finally, these unbiased DEGs were compared with DEGs identified in the original analysis for corresponding cell clusters to get a general result across PMN samples.

### Gene Ontology Enrichment Analyses

Gene Ontology functional analysis was performed using the R package clusterProfiler.^[^ ^59^ ^]^ Differentially expressed genes (DEGs) between PMN and Ctrl in multiple cell types were collected for subsequent enrichment analyses of functions and pathways by annotating Gene Ontology terms, including biological process, molecular function, and cellular component.

### Analyzing the Activation Process of B Cells and Mononuclear Phagocytes

Abnormal activation of immune cells was known to be associated with the pathogeny of PMN. In the study, pseudotime analysis was conducted by the R package Monocle2^[^ ^60^ ^]^ to reveal the activation process of B cells (except B cells from MN11) and monophagocytes, respectively. Highly variable genes among corresponding cell subtypes were identified by Seurat (v4.1.0).^[^ ^48^ ^]^ “DDRTree” algorithm built in the function “reduceDimension” was used to produce a 2D projection of the trajectory. Cells were then ordered along the pseudotime by “orderCells” function. The direction of the trajectory was set manually according to the expression level of known markers.

### Cell‐Cell Interaction Analysis

To investigate and comprehend the changes caused by PMN from the perspective of cell–cell communication, the R package CellChat^[^ ^61^ ^]^ was applied to infer and compare the total crosstalk in PMN and Ctrl. Based on the co‐expression level of ligand and receptor collected by cellchatDB,^[^ ^61^ ^]^ upregulated and downregulated receptor‒ligand pairings were predicted among glomerular cells and immune cells, which were closely bound to PMN pathogenesis.

It was further tested if a receptor‐ligand pair with significant signals was also enriched in either PMN cells or controls using an enrichment method^[^ ^62^ ^]^ Specifically, for each significant ligand‐receptor pair *i* (*i* ∈ *T*), the following table was constructed:

**Table:** 

	Pair ***i***	Pair *** t *** (***t*** ∈ ***T***, *** t *** ≠ ***i***)	Total
PMN	*a*	*m‐a*	*m*
Ctrl	*b*	*n‐b*	*n*

Where *a* and *b* were the frequency of pair *i* detected in cells of PMN and Ctrl, respectively. *m* and *n* were the frequency of all significant interactions *T* detected in cells of PMN and Ctrl, respectively.

The fold enrichment estimate was defined as:

(2) $$fold\;enrichment\;=\;\frac{a/m}{b/n}$$

The significance of the enrichment was measured by Fisher exact test.

Finally, the putative communications between various cell types stimulated by important ligand‐receptor pairings were visualized by bubble plots.

To avoid results biased by abnormal numbers of cells due to other etiologies, B cells from MN11 and NE from Ctrl6 were excluded from the prediction of cell‐cell communication.

### Cell Culture

Immortalized mouse podocytes (MPC‐5, Ximbio Cat#152 136, RRID: CVCL_AS87) were cultured in this study as previously reported.^[^ ^63^ ^]^ For growth permissive conditions, podocytes were cultured in Dulbecco's modified Eagle's medium (DMEM, HyClone) supplemented with 10% heat‐inactivated fetal bovine serum (Gibco), 20 U mL^−1^ mouse recombinant interferon‐γ (Peprotech), and 100 U ml^−1^ penicillin plus 100 µg ml^−1^ streptomycin (Sigma) at 33 °C; to induce differentiation, podocytes were maintained at 37 °C without interferon‐γ for 7 days. Differentiated podocytes were seeded onto 6‐well plates to reach a confluence of 70% and then starved in serum‐free DMEM overnight before experiments.

For BMP2 induction, cells were incubated with BMP2 recombinant protein (Novoprotein) at the indicated concentrations for the indicated durations. For complement stimulation, cells were incubated with 2 µg ml^−1^ mouse complement C3/C3a protein (MedChemExpress)^[^ ^17^ ^]^ or sublytic complement C5b‐9, assembled by 0.8 µg ml^−1^ human complement C5b6 protein (Sigma‒Aldrich) with 5% normal human serum^[^ ^17^, ^64^ ^]^ for the indicated durations.

### Immunofluorescence and Immunohistochemistry Staining

Immunofluorescence and immunohistochemistry staining were carried out on 4‐mm paraffin human biopsies. For immunolabeling of paraffin sections, heat‐induced antigen retrieval in antigen retrieval solution was carried out after deparaffinization and rehydration. Endogenous peroxidase activity was blocked in 3% H_2_O_2_ for 10 min for immunohistochemistry staining. The sections were then washed with PBS for 5 min and submerged in blocking reagent for 1 h at room temperature, followed by primary antibody incubation in blocking buffer at 4 °C overnight. Then, sections were stained with secondary antibodies. The primary antibodies were rabbit anti‐Wilms Tumor Protein (Abcam Cat#ab89901), rabbit anti‐BMP2 (Abcam Cat#ab284387), rabbit anti‐Smad1 (phospho‐S463 + S465) (Abcam Cat#ab214423), rabbit anti‐collagen IV (Abcam Cat#ab6586), and rabbit anti‐CD163 (Abcam Cat#ab182422). The secondary antibodies were Alexa Fluor conjugated antibodies for immunofluorescence or horseradish peroxidase‐conjugated antibodies for immunohistochemistry. Images were captured on a Zeiss Axio Vert A1 microscope and quantified with ImageJ.

### Western Blotting

Proteins from cultured podocytes were extracted using RIPA lysis buffer supplemented with a cocktail of protease and phosphatase inhibitors. The total protein concentration was measured using a BCA assay, and equal amounts of protein were loaded on PAGE 4%–20% gradient gels (GenScript) and transferred to PVDF membranes (Millipore). The primary antibodies were rabbit anti‐collagen IV (Abcam Cat#ab6586), rabbit anti‐Smad1 (phospho‐S463 + S465) (Abcam Cat#ab214423), rabbit anti‐Smad1 (D59D7) (Cell Signaling Technology Cat#6944), rabbit anti‐BMP2 (Abcam Cat#ab284387), and mouse anti‐β‐actin (Cell Signaling Technology Cat#3700). Blots were developed using Omni ECL reagent (EpiZyme).

### RNA Extraction and Real‐Time PCR

Total RNA from cultured podocytes was extracted using TRIzol Reagent (Invitrogen). RNA quality was checked by controlling the OD at 260 and 280 nm. cDNA was synthesized from 1 mg RNA using the Hifair II 1st Strand cDNA Synthesis Super Mix (Yeasen, China). Real‐time PCR was performed with the Roche Light Cycler 480 detection system using SYBR green PCR master Mix (Roche Diagnostics). Specific primers for target mRNAs, Col4a1 5′‐ CTGGCACAAAAGGGACGAG‐3′ and 5′‐ ACGTGGCCGAGAATTTCACC‐3′; Col4a2 5′‐ GACCGAGTGCGGTTCAAAG‐3′ and 5′‐ CGCAGGGCACATCCAACTT‐3′; Col4a3 5′‐CAAAGGCATCAGGGGAATAACT‐3′ and 5′‐ATCCGTTGCATCCTGGTAAAC‐3′; Col4a4 5′‐ATGAGGTGCTTTTTCAGATGGAC‐3′ and 5′‐GGGGCCGCCATACTTCTTG‐3′; Col4a5 5′‐GGAGAACGGGGGTTTCCAG‐3′ and 5′‐CTCCCTTGGTTCCATTGCATC‐3′; Bmp2 5′‐GGGACCCGCTGTCTTCTAGT‐3′ and 5′‐TCAACTCAAATTCGCTGAGGAC‐3′; and β‐actin 5′‐GGCTGTATTCCCCTCCATCG‐3′ and 5′‐CCAGTTGGTAACAATGCCATGT‐3′, were used for amplification, and a standard curve was generated for each targeted transcript. All relative expression levels were normalized to β‐actin expression, and the results were analyzed using the ΔΔCt method.

### Enzyme‐Linked Immunosorbent Assay

BMP2 levels in the cell culture medium supernatant were detected using a mouse BMP‐2 ELISA kit (Boster Bio) according to the manufacturer's instructions. Briefly, cells were cultured and treated with reagents in the 6‐well plates with 2 mL of cell culture medium in each well. When collecting the cell culture medium, the exact number of cells in each well was counted using trypan blue staining and a hemocytometer. The absorbance was measured at 450 nm on a Cytation 3 Cell Imaging Multi Mode Reader (BioTek). The ELISA results were normalized to 1 000 000 cells for each sample.

### Statistical Analysis

For experimental data, analysis was performed using GraphPad Prism software. Differences between two groups were analyzed using Student's two‐tailed t‐test, and differences between more than two groups were analyzed using a one‐way ANOVA test. Throughout the manuscript, n represents the number of patients/control subjects for kidney biopsy section analysis, and biological replicates for cell culture experiments. *P*‐values are presented as follows: *P < 0.05; **P < 0.01; ***P < 0.001; NS, not significant. The statistical details of experiments can also be found in the figure legends.

For bioinformatic data, changes in cell components between PMN and Controls were tested by general linear hypotheses for GLMM with R package lme4 and multcomp, and *P*‐value <0.05 was considered statistically significant. Differentially activated pathways were identified by testing their GSVA scores in PMN and Ctrl cells, performed by Limma, and a *P*‐value <0.05 was considered statistically significant. Differentially expressed genes were identified using the function “FindMarkers” in Seurat (v4.1.0) with the “MAST” method, and FDR was used for multi‐test correction. Genes with FDR <0.05 and log(fold change) ≥0.3 were identified as significantly upregulated genes in PMN, while genes with FDR <0.05 and log(fold change) ≤−0.3 were determined to be significantly downregulated genes in PMN.

### Patient Consent Statement

The patient described in this study consented under the ethics committee review of Renji Hospital affiliated to Shanghai Jiao Tong University School of Medicine, and written informed consent was received prior to participation.

## Conflict of Interest

The authors declare no conflict of interest.

## Author Contributions

A.C., Y.M., and H.Z. contributed equally to this work. S.M. and L.C. designed the study. A.C., H.Z., H.C., X.S., Q.W., Y.X., Y.Z., W.Z., and S.M. collected patient samples and clinical data, and processed patient samples. A.C., Y.M., and H.Z. performed the sequencing and interpreted the data. A.C., Y.M., H.Z., H.C., L.C., and S.M. analyzed the data. A.C. performed in vitro and in vivo experiments. A.C. and Y.M. wrote the manuscript with contributions from all the authors. L.C. and S.M. revised the manuscript. All authors read and approved the final manuscript.

## Supporting information

Supporting Information

## Data Availability

The data that support the findings of this study are openly available in National Omics Data Encyclopedia at https://www.biosino.org/node/project/detail/OEP004186, reference number 4186.
