# Supplementary material for: Podocyte Pathogenic Bone Morphogenetic Protein‐2 Pathway and Immune Cell Behaviors in Primary Membranous Nephropathy
Source: Adv Sci (Weinh). 2024 May 24;11(29):2404151. doi: 10.1002/advs.202404151 (PMC11304328; doi:10.1002/advs.202404151)
Supplement: Supplementary file 1 — Supporting Information [file ADVS-11-2404151-s001.pdf]

ADVANCED SCIENCE
Open Access

Supporting Information

for *Adv. Sci.*, DOI 10.1002/advs.202404151

Podocyte Pathogenic Bone Morphogenetic Protein-2 Pathway and Immune Cell Behaviors in Primary Membranous Nephropathy

Anxiang Cai, Yiwei Meng, Hang Zhou, Hong Cai, Xinghua Shao, Qin Wang, Yao Xu, Yin Zhou, Wenyan Zhou, Luonan Chen and Shan Mou**

Supporting Information

Podocyte Pathogenic Bone Morphogenetic Protein-2 Pathway and Immune Cell Behaviors in Primary Membranous Nephropathy

*Anxiang Cai[1#], Yiwei Meng[2,3#], Hang Zhou[1#], Hong Cai[1], Xinghua Shao[1], Qin Wang[1], Yao Xu[1], Yin Zhou[1], Wenyan Zhou[1], Luonan Chen[2,4,5]** and Shan Mou[1]**

Supplemental Methods

Analysis of the potential tipping state of PMN podocytes by DNB

We supposed that the podocytes from PMN patients were still at different states shifting along PMN progression and we wondered if a cellular state is essential for PMN evolution. We first performed trajectory analysis on 114 podocytes of PMN patients by the R package monocle2[1] to obtain pseudo-states of cells shifting along PMN evolution. A standard workflow was employed to cluster cells and identify differentially expressed genes among cell clusters. The function 'reduceDimension' with the 'DDRTree' method was applied to reduce the dimensions, and the function 'orderCells' was used to order the cells. We further defined 3 subcellular states (or cell subtypes) according to the trajectory results and the expression levels of genes relative to PMN, from state 1 to state 3, podocytes were getting abnormal gradually (Figure S3D). Then we optimized our previous mathematical model, PDM algorithm,[2] to detect the potential tipping point/state of PMN podocytes. Theoretically, the PDM method is an extension of the dynamic network biomarkers (DNB) theory,[3] which can identify the tipping point or critical state, which is considered a time point just before the initiation or the critical transition during the progression of a certain disease; at/near the tipping point before the transition from normal (control) state to the disease state, a DNB module, also called a PDM, i.e. a number of genes/molecules will exhibit a particularly strong collective fluctuation, or called "critical collective fluctuation" (CCF, a higher order statistic), rather than traditional differential expressions of genes (the first order statistic). Such CCFs on DNB genes can be measured by higher-order statistics, e.g. differential distributions of gene expressions. A gene module with its functional interactions of TF-target regulations, known as a regulon, was constructed by prior knowledge and downloaded from the public database SwissRegulon

(https://swissregulon.unibas.ch/sr/). And based on the key finding that BMP2 is an important molecular, the activation of which can lead to GBM thickening in PMN, the regulons with BMP2 as a target were tested as candidate DNB modules. Given the expression matrix of each regulon at each state, we first reduced it to two-dimensional space by PCA (Principal Component Analysis). Next, based on KL divergence, we introduced $kl-Score$ to quantify the differential distribution of each tested regulon between cells in state i and in state 0 (controls) at the level of distribution, which was defined as

$$kl-Score_k = \frac{KL(P(X_i)\|P(X_0)}{\log(n_i \times n_0)}$$

where $P(X_i)$ and $P(X_0)$ are the joint probability density/distribution functions for the dimension-reduced expression data X_i and X_0 of regulon k in cells of the state i and cells of state 0 (controls), respectively, n_i and n_0 are the numbers of cells of state i and controls. As a DNB/PDM is supposed to change significantly in terms of distributions rather than mean expressions, it is necessary to adjust the $kl-Score$ by the difference of mean values between the two states, to reduce the influence of changes in means upon changes in distributions. Assuming that $P(X_i)$ and $P(X_0)$ follow multivariate normal distributions, then the difference in means could be quantified by $Hotelling\,T^2$ statistic:

$$T^2 = (\bar{x}_i - \bar{x}_0)^T \left[\left(\frac{1}{n_i} + \frac{1}{n_0}\right)S\right]^{-1} (\bar{x}_i - \bar{x}_0)$$

$$= \frac{1}{\frac{1}{n_i} + \frac{1}{n_0}} (\bar{x}_i - \bar{x}_0)^T \left[\frac{1}{n_i + n_0 - 2}\left((n_i - 1)S_i + (n_0 - 1)S_0\right)\right]^{-1} (\bar{x}_i - \bar{x}_0)$$

in which $\bar{x}_i, S_i$ and $\bar{x}_0, S_0$ are separately sample means and sample covariance matrices of X_i and X_0. T^2 was calculated by function “T2.test” in R package “rrcov”.

Thus, following the quantification index, an adjusted $kl-Score$, was utilized as the indicative signal of the PDM method:

$$adjusted\ kl - Score_k = \frac{KL(P(X_i)\|P(X_0))/T^2}{\log(n_i \times n_0)}$$

When the adjusted kl-Score of a molecular module reaches a peak at the measured state compared to the control state, the cells can be considered to be at a tipping state. And top 3 modules with the largest adjusted kl-Scores are regarded to constitute the PDMs of PMN.

The dynamic networks constructed by the tested regulons were profiled for each cell state by Cytoscape.[4] The functional enrichment analysis was implemented by the function "STRING Enrichment" of the application "stringApp" built in Cytoscape [5].

Analysis of published scRNA-seq data of IgAN

To investigate whether BMP2/pSMAD1/COL4 pathway is specific to PMN, we collected two published scRNA-seq profiles of human renal tissue from IgAN patients and the paired controls (HRA000342 and GSE171314), and analyzed each profile separately because different experimental conditions and sequencing instrument models could lead to the variation of sequencing depth as well as severe batch effects. For each profile, cell clustering and annotation were the same as in the original research, DEGs between IgAN and control sets were identified for podocytes and HRMC using the "MAST" method implemented by the "FindMarkers" function in R package Seurat (v4.1.0) and were further performed GO enrichment analysis. Additionally, expression levels of BMP2 and COL4 in podocytes and HRMC were drawn and compared between IgAN and control samples by violin plots.

Testing the role of C3aR and C5aR in complement-induced BMP2 pathway

To investigate the expression of C3aR and C5aR after C3a or sublytic complement C5b-9 incubation for 48 hours, MPC-5 cells were treated with complement components as previously described, and cell lysates were collected and analyzed using Western blotting. The primary antibodies were mouse

anti-C3aR (Santa Cruz Biotechnology, CATALOG # sc-133172) and mouse anti-C5aR (Santa Cruz Biotechnology, CATALOG # sc-53797).

For inhibitor experiments, cells were incubated with C3a+C3aR inhibitor SB290157 (10µM, MedChemExpress) or C5aR inhibitor PMX-53 (1µM, MedChemExpress), or C5b-9+C3aR inhibitor SB290157 or C5aR inhibitor PMX-53, for 48 hours. Cell lysates were then collected and analyzed using Western blotting as previously described.

Supplemental Results

Random sub-sampling to identify unbiased DEGs

Random sub-sampling was implemented for 100 times to identify unbiased DEGs between PMN and control samples for cell sub-types consisting of cells mostly from one or two individuals. These cell types with the dominant subjects were listed in Supplemental Table S5. Note that none of the subjects had an overwhelming number of podocytes, indicating that changes in podocytes were common in most of our PMN patients. For G-Endo, monocytes, macrophages, $CD4^{+}$Th, and $CD8^{+}$CTL, unbiased DEGs identified based on random sub-sampling were found to largely overlap with DEGs detected by regular tests we initially applied to differential gene analysis, and the overlap proportion of the top 10% of DEGs (ranked by FDR) increased (Figure S1C). Afterward, all the common (unbiased) DEGs were used for enrichment analysis of GO terms, reproducing a majority of up and down-regulated functions in the original GO enrichment results (Figure S1D). Moreover, expression distributions of important differential genes in podocytes, M2-like macrophages, memory B cells, and plasmas were depicted for each PMN and control subject, showing that key genes expressed in crucial cell types associated with PMN changed consistently in the majority of PMN patients (Figure S3A, 4E, 5F). Consequently, despite the observation that B cells lacked consistency among the majority of PMN patients mainly because B cells were not obtained in many renal biopsies, the main changes in podocytes, myeloid immune cells, and T cells were more likely to be general across the PMN patients.

Identification of the potential tipping state of PMN podocytes by DNB

Cells, organisms, and human bodies are dynamic homeostasis systems. When a disease occurs, the

biosystems upset the intrinsic balance to establish a new one, and the 'tipping state' is considered as a time point when the biological system is about to tip the equilibrium, i.e., the state just before the initiation or the critical transition during the progression of a certain disease.[3] The detection and investigation of tipping state hold the key to early warning and early treatment of the disease. To investigate the possible transition of podocytes during PMN occurrence, we performed the trajectory analysis on 114 podocytes of PMN patients and defined 3 subcellular states along pseudotime. From cell state 1 to 3, *BMP2*, *COL4*, and HLA class I were upregulated while *CRYAB* and *AIF1L* were downregulated gradually (Figure S3D). In order to predict a cellular state that is a tipping state for PMN evolution, our previous potential disease module (PDM) algorithm[2] based on the dynamic network biomarkers (DNB) theory[3] was employed to identify the potential tipping state (pre-malignant state) from the 3 states of podocytes inferred by pseudotime analysis. As shown in the dynamic networks during the measured periods, a strong signal of the tipping state was found at state 2, where adjusted kl-Scores of most BMP2-related gene modules (regulons) reached the highest values based on the PDM model, indicating that cell state 2 was a potential critical state (Figure S3E). According to functional enrichment results, the top 3 leading modules at the tipping state markedly changed in phosphorylation, protein folding, TGF-beta signaling pathways, and pathways related to the immune system, indicating a significant relationship between podocytes in cell state 2 and PMN evolution through ECM production triggered by *BMP2* expression (Figure S3F).

Renal fibroblast activities in PMN

Of note, fibroblasts, a key inducer of renal fibrosis via producing multiple fibrotic cytokines and

ECM,[6] presented a higher proportion in PMN renal interstitial cells (mean percentage as 5.52% in PMN versus 2.20% in Ctrls, P=0.051, Figure 2B, Supplemental Table S3). Nevertheless, in our data, fibroblasts did not actively upregulate fibrotic genes or pathways: even though upregulated pathways were enriched in ribosome activity, their production of ECM components was even downregulated (Figure S3G-H), which disapproved its role in GBM thickening and on the contrary highlighted the importance of podocytes to the histopathology of PMN.

Role of C3aR and C5aR in complement-induced BMP2/pSMAD1/COL4 signaling pathway

Normally, ligands bind their receptors to exert their effects. C3aR and C5aR are two cognate receptors for C3a and C5a that initiate maladaptive pathways in PMN podocytes. [7] We tested their roles in our identified complement-induced BMP2/pSMAD1/COL4 signaling pathway. C3a incubation increased C3aR expression in podocytes but showed no effect on C5aR levels; whereas C5b-9 incubation did not impact the expression of C3aR or C5aR (Figure S5A). We then utilized C3aR inhibitor SB290157 and C5aR inhibitor PMX-53 to investigate the involvement of these two receptors in SMAD1 phosphorylation and collagen IV production. Blocking C3aR inhibited C3a-induced SMAD1 phosphorylation and collagen IV production, while C5aR inhibition failed to attenuate this process (Figure S5B). In contrast, SB290157 and PMX-53 seemed to have no significant effect on C5b-9-induced SMAD1 phosphorylation and collagen IV production (Figure S5C). According to these results, C3aR rather than C5aR participated in the C3a-induced BMP2 pathway, while the C5b-9-induced BMP2 pathway was independent of C3aR or C5aR.

Renal tubular cells in PMN

Renal tubular cells are not the primary targets of PMN but may be secondarily impacted by podocyte injury. Tubular cells in the current study were subclustered as PT with a small cluster expressing *VCAM1* (PT-*VCAM1*$^{+}$), LOH, DT, CD-PC, and CD-IC (Figure S9A-D). No significant change in tubular component percentages was observed (Figure S9E). Previous studies have reported that *VCAM1*$^{+}$ proximal tubular cells could activate the NF-κB, TNF, and AP-1 signaling pathways in response to kidney injury.[8] However, we observed apparently augmented expression of only two interferon-inducible genes, namely, *IFI27* and *IFI6* (data not shown). GO analysis suggested elevated antigen presentation and ECM synthesis in PT-*VCAM1*$^{+}$ and LOH (Figure S9F). Nevertheless, no other specific information concerning the pathogenesis of PMN in this group of cells was provided.

Comparison between PMN patients with low and high proteinuria levels

To reveal potential mechanisms for disease progression, we compared patients with low and high proteinuria levels, exploring their transcriptomic differences in podocytes and other renal intrinsic and immune cells. In our scRNA-seq cohort, 5/11 patients have a level of proteinuria > 3000mg/24h, and 6/11 patients < 3000mg/24h. Patients of these two groups had significant differences in their 24h proteinuria levels (1914.9±771.7 versus 5910.2±3010.6 mg, *P*=0.0197), but are comparable in age (58.7±5.5 versus 58.6±11.0 years, *P*=0.9909), serum creatinine (60.5±11.3 versus 75.0±19.8 μM, *P*=0.2021), and estimated glomerular filtration rate (97.2±10.6 versus 80.8±22.2 ml/min/1.73 m2, *P*=0.1819). Cell percentages of major cell types presented no significant differences between patients with low and high proteinuria levels (data not shown).

For glomerular cells, GSVA analysis revealed protein chromophore, phagocytosis, humoral immune

response, and complement system activation as the major upregulated pathways in podocytes of the high proteinuria group; while NOD pathway, nuclease activity, and regulation of inflammation were major downregulated pathways (Figure S10A). Although both patient groups displayed augmented levels of *BMP2*, *COL4A3*, and *COL4A4* compared with the Ctrl group, no significant changes were observed between patients with low and high proteinuria levels (Figure S10B). Moreover, PECs in the high proteinuria group upregulated pathways including cell differentiation, complement activation, phagocytosis, and respiration; downregulated pathways involved left-right asymmetry and fatty acid metabolism (Figure S10C). In addition, glomerular endothelium showed increased phagocytosis, humoral immune response, and complement activation; and decreased pathways mainly focused on NOD and NOD2 signaling pathways (Figure S10D). Mesangial cells from these two groups had few DEGs and no pathway enrichment results.

For other cell types, GO analysis unraveled elevated unspecific pathways of cytochrome, mitochondrial respiration, and mRNA binding of myeloid leukocytes, and M2-like Mpa majorly upregulated mitochondrial and ribosome pathways (Figure S10E). In B cells, only plasma cells presented differences between two patient groups, involving augmented cell respiration and vesicule activity (Figure S10F). Besides, among renal interstitial cells, fibroblasts upregulated cell-substrate junction organization, proteasome activity, and nuclear speck pathways (Figure S10G). Furthermore, in the high proteinuria group, Th and CTL cells had more active antigen receptor signal transduction and B cell activation process; Tregs activated B cell receptor; NK cells upregulated immune response, phagocytosis, splicesome, and immunoglobin pathways (Figure S10H).

We noticed that the expression levels of BMP2/COL4 seemed not to be directly associated with the amount of proteinuria in early-stage PMN patients, whereas humoral immune response and

complement activation pathways were more severe in high proteinuria patients. Also, humoral immune response and complement activation pathways were more severe in PECs and G-Endos from high proteinuria patients, and these patients had more active myeloid leukocytes, plasma cells, and T and NK cells in their diseased kidneys, implying an important role of immunoglobin-induced complement-mediated inflammation in the amount of proteinuria and PMN progression.

Overlapped DEGs among different cell types

Of interest, there were overlaps of several genes with altered expression levels among different cell types. We observed downregulated *HSPs* in multiple cell clusters from PMN patients. HSPs are molecular chaperones, and recent studies disclosed their roles in modulating the immune system: either promoting or inhibiting inflammation depending on their concentrations and participating in antigen presentation.[9] Future investigations should answer whether HSP downregulation in PMN is causal or consequential, protective or detrimental. Moreover, the NOD and NOD2 pathways were downregulated in patients' B lymphocytes and glomerular cells. NODs are responsible for sensing bacterial infection and inducing proinflammatory responses; they participate in intestinal homeostasis, and their dysfunction is associated with inflammatory bowel diseases.[10] We reported decreased NOD activity in PMN, expanding their involvement in renal autoimmune diseases. Additionally, we detected elevated *HLA-DQA2* expression in two major APCs, memory B cells and M2-like macrophages. Various investigations have identified HLA-DQA1, HLA-DRB1, and HLA-DRB3 as risk alleles for PMN,[11] while this study might provide new information on the association between HLA-DQA2 and PMN.

References

[1] X. J. Qiu, A. Hill, J. Packer, D. J. Lin, Y. A. Ma, C. Trapnell, *Nature Methods* **2017**, *14* (3), 309, https://doi.org/10.1038/Nmeth.4150.

[2] Y. Meng, Y. Huang, X. Chang, X. Liu, L. Chen, *Brief Bioinform* **2022**, *23* (2), https://doi.org/10.1093/bib/bbab608.

[3] a) L. Chen, R. Liu, Z. P. Liu, M. Li, K. Aihara, *Sci Rep* **2012**, *2*, 342, https://doi.org/10.1038/srep00342; b) Y. Zhang, C. Zuo, L. Liu, Y. Hu, B. Yang, S. Qiu, Y. Li, D. Cao, Z. Ju, J. Ge, Q. Wang, T. Wang, L. Bai, Y. Yang, G. Li, Z. Shao, Y. Gao, Y. Li, R. Bian, H. Miao, L. Li, X. Li, C. Jiang, S. Yan, Z. Wang, Z. Wang, X. Cui, W. Huang, D. Xiang, C. Wang, Q. Li, X. Wu, W. Gong, Y. Liu, R. Shao, F. Liu, M. Li, L. Chen, Y. Liu, *J Hepatol* **2021**, *75* (5), 1128, https://doi.org/10.1016/j.jhep.2021.06.023.

[4] K. Ono, T. Muetze, G. Kolishovski, P. Shannon, B. Demchak, *F1000Res* **2015**, *4*, 478, https://doi.org/10.12688/f1000research.6767.1.

[5] N. T. Doncheva, J. H. Morris, J. Gorodkin, L. J. Jensen, *J Proteome Res* **2019**, *18* (2), 623, https://doi.org/10.1021/acs.jproteome.8b00702.

[6] Y. Liu, *Nat Rev Nephrol* **2011**, *7* (12), 684, https://doi.org/10.1038/nrneph.2011.149.

[7] P. Ronco, L. Beck, H. Debiec, F. C. Fervenza, F. F. Hou, V. Jha, S. Sethi, A. Tong, M. Vivarelli, J. Wetzels, *Nat Rev Dis Primers* **2021**, *7* (1), 69, https://doi.org/10.1038/s41572-021-00303-z.

[8] a) L. M. S. Gerhardt, J. Liu, K. Koppitch, P. E. Cippà, A. P. McMahon, *Proc Natl Acad Sci U S A* **2021**, *118* (27), https://doi.org/10.1073/pnas.2026684118; b) Y. Muto, P. C. Wilson, N. Ledru, H. Wu, H. Dimke, S. S. Waikar, B. D. Humphreys, *Nat Commun* **2021**, *12* (1), 2190, https://doi.org/10.1038/s41467-021-22368-w.

[9] a) T. Zininga, L. Ramatsui, A. Shonhai, *Molecules* **2018**, *23* (11), https://doi.org/10.3390/molecules23112846; b) S. Tukaj, *Int J Mol Sci* **2020**, *21* (15), https://doi.org/10.3390/ijms21155298.

[10] a) R. Caruso, N. Warner, N. Inohara, G. Núñez, *Immunity* **2014**, *41* (6), 898, https://doi.org/10.1016/j.immuni.2014.12.010; b) D. J. Philpott, M. T. Sorbara, S. J. Robertson, K. Croitoru, S. E. Girardin, *Nat Rev Immunol* **2014**, *14* (1), 9, https://doi.org/10.1038/nri3565.

[11] a) H. C. Stanescu, M. Arcos-Burgos, A. Medlar, D. Bockenhauer, A. Kottgen, L. Dragomirescu, C. Voinescu, N. Patel, K. Pearce, M. Hubank, H. A. Stephens, V. Laundy, S. Padmanabhan, A. Zawadzka, J. M. Hofstra, M. J. Coenen, M. den Heijer, L. A. Kiemeney, D. Bacq-Daian, B. Stengel, S. H. Powis, P. Brenchley, J. Feehally, A. J. Rees, H. Debiec, J. F. Wetzels, P. Ronco, P. W. Mathieson, R. Kleta, *N Engl J Med* **2011**, *364* (7), 616, https://doi.org/10.1056/NEJMoa1009742; b) W. B. Le, J. S. Shi, T. Zhang, L. Liu, H. Z. Qin, S. Liang, Y. W. Zhang, C. X. Zheng, S. Jiang, W. S. Qin, H. T. Zhang, Z. H. Liu, *J Am Soc Nephrol* **2017**, *28* (5), 1642, https://doi.org/10.1681/asn.2016060644; c) Z. Cui, L. J. Xie, F. J. Chen, Z. Y. Pei, L. J. Zhang, Z. Qu, J. Huang, Q. H. Gu, Y. M. Zhang, X. Wang, F. Wang, L. Q. Meng, G. Liu, X. J. Zhou, L. Zhu, J. C. Lv, F. Liu, H. Zhang, Y. H. Liao, L. H. Lai, P. Ronco, M. H. Zhao, *J Am Soc Nephrol* **2017**, *28* (5), 1651, https://doi.org/10.1681/asn.2016020114.

Supplemental Tables

Supplemental Table S1. Marker genes of different cell types

Cell type	Abbreviation	Marker gene
Podocytes	Podo	$WT1^{+}$, $NPHS1^{+}$, $NPHS2^{+}$
Glomerular endothelial cells	G-Endo	$PECAM1^{+}$, KDR^{+}, $EHD3^{+}$
Peritubular endothelial cells	T-Endo	$PECAM1^{+}$, KDR^{+}
Arterial endothelial cells	A-Endo	$PECAM1^{+}$, KDR-, $SEMA3G^{+}$
Human renal mesangial cells	HRMC	$GATA3^{+}$, $PDGFRB^{+}$
Vascular smooth muscle cells	VSMC	$MUSTN1^{+}$, $ACTA2^{+}$
Glomerular parietal epithelial cells	PEC	CLDN1, $PAX8^{+}$
Fibroblasts	Fibro	LUM^{+}, DCN^{+}
Naïve B cells	Naïve B	$CD19^{+}$, CD27-
Memory B cells	Memory B	$CD19^{+}$, $CD20^{+}$, $CD27^{+}$
Plasma cells	Plasma	CD19-, $CD27^{+}$, $CD138^{+}$
$CD4^{+}$ helper T cells	$CD4^{+}$ Th	$CD3^{+}$, $CD4^{+}$, CD8-
Regulatory T cells	Treg	$CD4^{+}$, $CD25^{+}$, $FOXP3^{+}$
$CD8^{+}$ cytotoxic T cells	$CD8^{+}$ CTL	$CD3^{+}$, CD4-, $CD8^{+}$
Natural killer cells	NK	$KLRD1^{+}$, $NKG7^{+}$
Neutrophils	NE	$FCGR3B^{+}$, $MNDA^{+}$
Nonclassical Monocytes	Nonclassical Mono	$CD68^{+}$, CD14-, $CD16^{+}$
Classical Monocytes	Classical Mono	$CD68^{+}$, $CD14^{+}$, CD16-
type 2 conventional dendritic cells	cDC2	$FCGR2B^{+}$, $CD1C^{+}$
M2-like macrophages	M2 like MPA	$CD68^{+}$, $CD163^{+}$, $MRC1^{+}$
Lipid-associated macrophages	Lipid-associated MPA	$CD86^{+}$, $TREM2^{+}$
Mast cells	Mast	$TPSB2^{+}$, $CPA3^{+}$
Proximal tubular cells	PT	$MIOX^{+}$, $SLC22A8^{+}$
$VCAM1^{+}$ proximal tubular cells	PT_$VCAM1^{+}$	$VACM1^{+}$, $MIOX^{+}$, $SLC22A8^{+}$
Loop of Helen cells	LOH	$SLC12A1^{+}$
Distal tubular cells	DT	$SLC8A1^{+}$, $SLC12A3^{+}$
Collecting duct-intercalated cells	CD-IC	$ATP6V0D2^{+}$

Collecting duct-principal cells	CD-PC	$AQP2^{+}$

Supplemental Table S2. The number of each cell compartment and each major cell type

Cell compartment	The number of cells	Cell type	The number of cells
**Tubule**	53061	**PT**	41744
		**PT-VCAM1$^+$**	3154
		**DT & LOH**	3970
		**CD-PC**	955
		**CD-IC**	3238
**Glomerular & interstitial cells**	8972	**Endo**	6996
		**HRMC**	989
		**Podo & PEC**	515
		**Fibroblast**	472
**T & NK cell**	5088	**T cell**	3558
		**NK cell**	1530
**Myeloid leukocyte**	3677	**Mono & Mpa**	2692
		**NE**	985
**B lymphocyte**	1576	**B cell**	1576

Supplemental Table S3. The number of each cell compartment and each cell sub-type

Cell compartment	The number of cells	Cell sub-type	The number of cells	PMN	Ctrl
**Tubule**	53061	**PT**	43165	28140	15025
		**PT-VCAM1^{+}**	2126	991	1135
		**LOH**	2769	1804	965
		**CD-IC**	2718	1644	1074
		**CD-PC**	1719	1171	548
		**DT**	564	393	171
**Glomerulus & renal interstitial cells**	8972	**T-Endo**	4645	1989	2656
		**G-Endo**	1520	240	1280
		**A-Endo**	485	152	333
		**Podocyte**	295	181	114
		**PEC**	200	157	33
		**Fibroblast**	356	304	52
		**VSMC**	1291	666	625
		**HRMC**	180	51	129
**T & NK cells**	5088	**CD4^{+} Th**	1707	1155	552
		**CD8^{+} CTL**	2161	1574	587
		**NK**	1052	379	673
		**Treg**	143	123	20
		**Proliferating T**	25	16	9
**Myeloid leukocytes**	3677	**cDC2**	226	153	73
		**Classical mono**	407	127	280
		**Non-classical mono**	543	249	294
		**Intermediate Mpa**	202	135	67
		**M2-like Mpa**	540	411	129
		**Trem2^{+} Mpa**	375	233	142
		**Proliferating Mpa**	26	17	9
		**NE**	1227	155	1072
		**Mast**	131	100	31
**B lymphocytes**	1576	**Memory B**	218	90	128
		**Naïve B**	38	28	10
		**Plasma**	163	142	21
		**B cell (MN11)**	1157	**NA**	

Note:

* For clustering cells more accurately, sub-clustering was performed within each cell compartment to amend the mis-clusters that might occur in the first-step clustering. Therefore, it is reasonable for the inconsistency between cell type and cell sub-type.

* The patient sample MN11 had abnormal counts of B cells, which were excluded in the sub-clustering and analysis of B lymphocytes to avoid sample-specific effects

Supplemental Table S4. The mean and median percentage of each cell sub-type in PMN versus Ctrl

Cell category	Cell subtype	Mean percentage (%)		Median percentage (%)		*P*-value
		PMN	Ctrl	PMN	Ctrl	
Glomerular & interstitial cells	Podocytes	24.80494828	17.92408497	15.625	16.66666667	0.543435288
	PEC	28.62186831	2.672628903	27.23735409	1.153212521	5.98E-07
	G-Endo	38.98538774	70.53778073	30.43478261	70.3125	4.89E-06
	HRMC	7.587795665	8.86550539	4.545454545	7.042253521	0.468508714
	A-Endo	7.236776378	14.40654599	7.37704918	15.18987342	0.005435935
	T-Endo	68.68523364	68.97147194	64.9122807	70	0.729980897
	Fibroblasts	5.516993641	2.203591822	3.03030303	1.25	0.051414481
	VSMC	18.56099634	14.41839025	16.12903226	15.09433962	0.230179401
B cells	Memory B	41.29657444	82.00061843	40.03623188	85.71428571	0.005408794
	Plasma	48.70342556	17.99938157	49.35770751	14.28571429	0.005408444
Myeloid leukocytes	cDC2	8.154430192	5.602881418	7.096774194	4.533678756	0.350532069
	Classical Mono	9.274842581	18.33542106	7.567567568	14.63414634	0.191435837
	Nonclassical Mono	19.75614045	16.82426185	13.28125	15	0.749269013
	Intermediate Mpa	13.51285294	6.404695229	10.71428571	2.720207254	0.086435415
	M2-like_Mpa	23.04904426	8.933120623	22.58064516	9.090909091	0.031080415
	$TREM2^{+}$Mpa	12.00818041	19.51684208	14.88095238	19.04761905	0.515278922
	NE	8.154430192	6.282484629	7.096774194	5.925375964	0.689155177
	Mast	4.359783325	0.988398007	3.243243243	0	0.073165899
T & NK cells	$CD8^{+}$CTL	48.92077487	31.40314889	47.71048744	32.05882353	0.006053849
	$CD4^{+}$Th	28.92918183	37.87490519	29.05027933	32.3943662	0.352454855
	Treg	3.372189149	0.758221456	3.149606299	0	0.000266393
	NK	18.77785416	29.96372447	20.63492063	35.16483516	0.02080227
Tubule	PT	76.68201793	63.92316375	89.41967445	79.38177699	0.131103939
	$PT\text{-}VCAM1^{+}$	3.794699252	4.841119652	3.157894737	4.767419417	0.214077496
	LOH	6.155259451	7.215157242	2.972399151	5.227383072	0.321906124
	DT	1.485447801	1.208568438	0.778485492	1.229972487	0.673021384
	CD-PC	4.519536554	8.200340225	1.606824043	3.49127182	0.310519627
	CD-IC	7.363039011	14.61165069	2.201944059	5.583427739	0.277648874

Supplemental Table S5. Cell types in which most cells were from certain subjects

Cell type	Dominant subject
G-Endo	MN10
HRMC	MN10
PEC	MN10, Ctrl5
Memory B	MN10, Ctrl5
Plasma	MN8, MN10, Ctrl5
$CD4^+$ Th	MN11
$CD8^+$ CTL	MN11
Treg	Ctrl5
Classical Mono	Ctrl5
Nonclassical Mono	Ctrl5
M2-like Mpa	MN10
$TREM2^+$ Mpa	MN10
Mast	MN8, Ctrl5
NE	MN2, Ctrl6

Supplemental Figures and Figure Legends

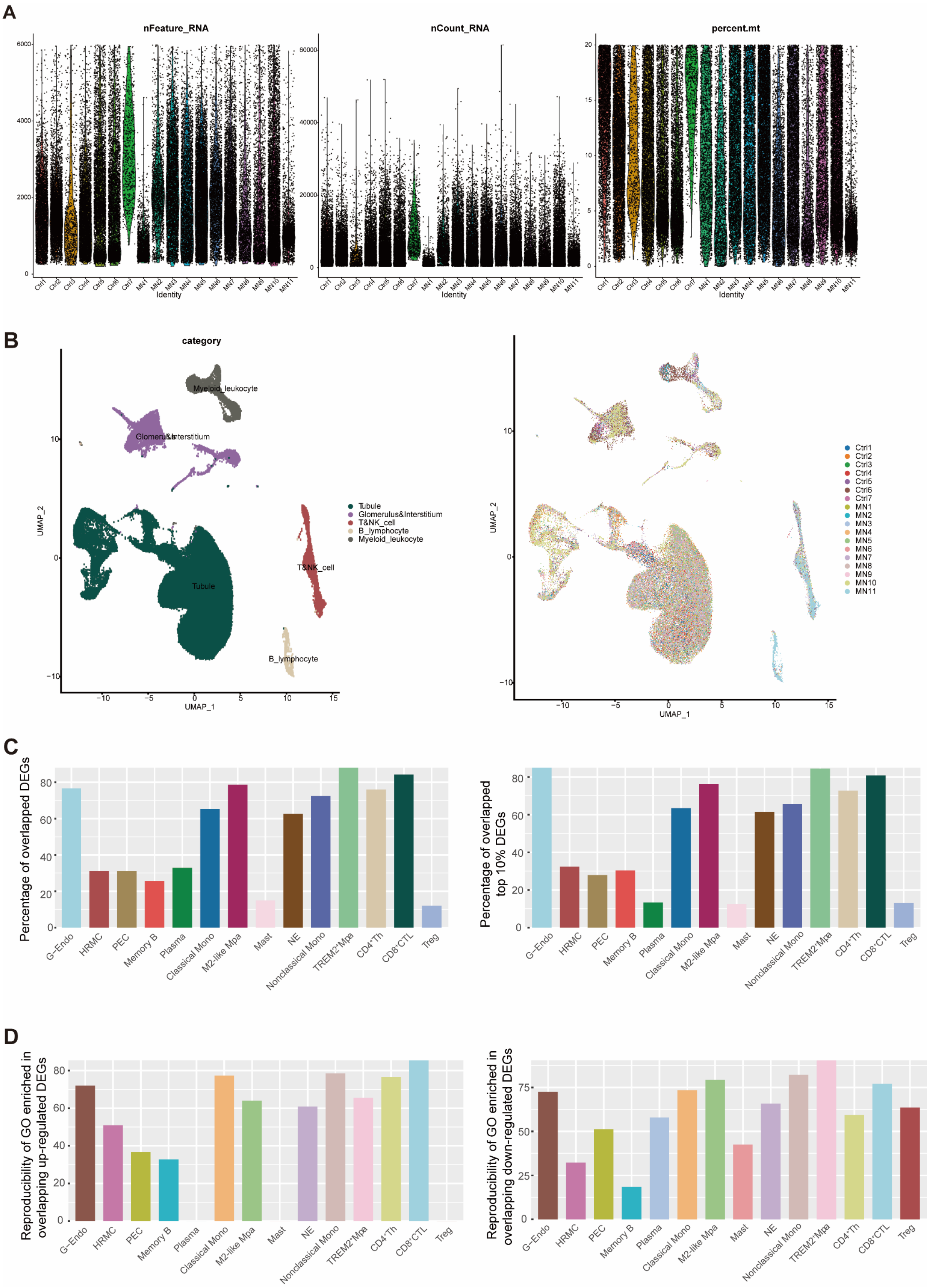

Supplemental Figure S1. Single-cell RNA sequencing quality control metrics

A. ScRNA-seq quality control data showing the number of genes detected in each cell (left), the total number of molecules detected in each cell (middle), and the percentage of mitochondrial genes in each cell (right). **B.** UMAP visualization of the clustering of 72 374 cells in total, color-coded by different cell categories. (left) UMAP visualization of all cells from each sample. (right) **C.** Percentages of overlap between the total (left) and top 10% (right) differential genes identified based on random sub-sampling and those found by the routine tests. **D.** Percentages of GO terms reproduced by enrichment analysis of up- (left) and down- (right) regulated differential genes both identified based on random sub-sampling and regular tests.

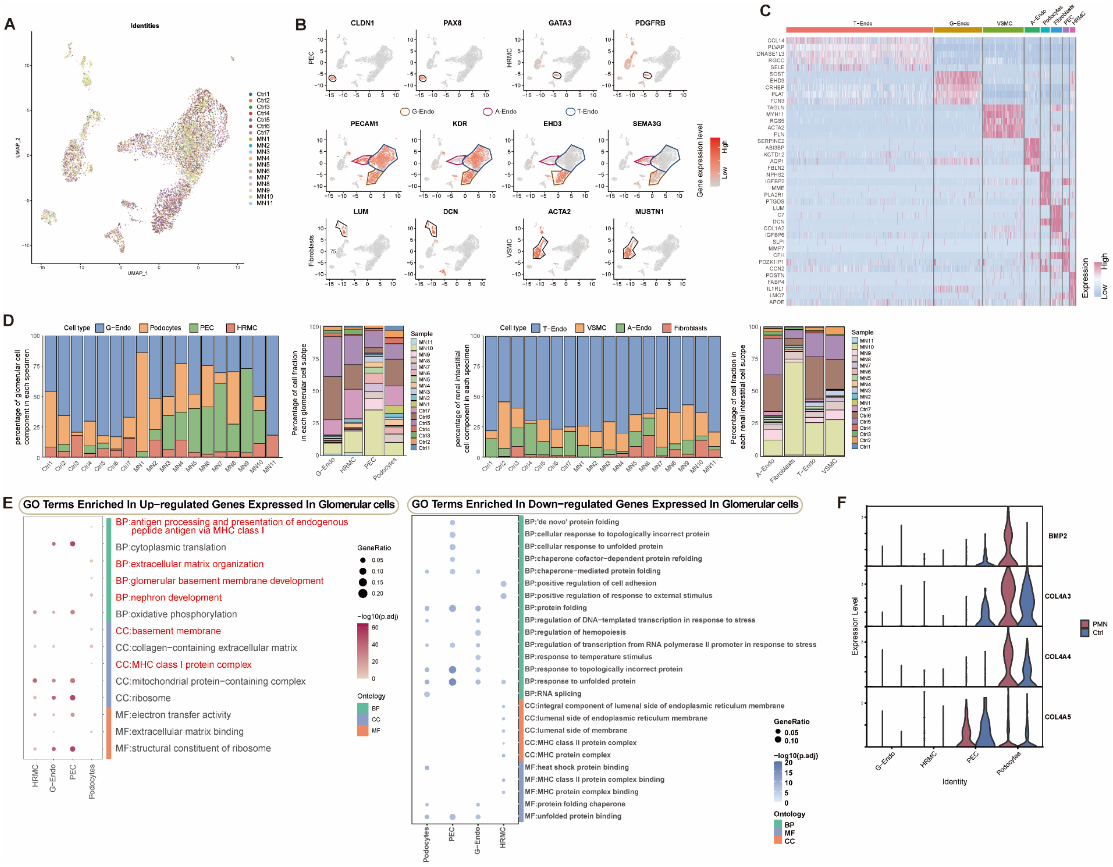

Supplemental Figure S2. Renal glomerular and interstitial cell alterations in PMN

A. UMAP visualization of glomerular and interstitial cells from each sample. **B.** Expression distribution of marker genes of different glomerular and interstitial cell types. **C.** Heatmap indicating distinct gene expression patterns of different glomerular and interstitial cell types. **D.** Percentages of different glomerular and interstitial cell types from each sample. **E.** GO analysis presenting major upregulated and downregulated pathways of HRMCs, G-Endos, PECs, and podocytes. **F.** Violin plot showing the expression levels of *BMP2*, *COL4A3*, *COL4A4*, and *COL4A5* in HRMCs, G-Endos, PECs, and podocytes from PMN versus Ctrl.

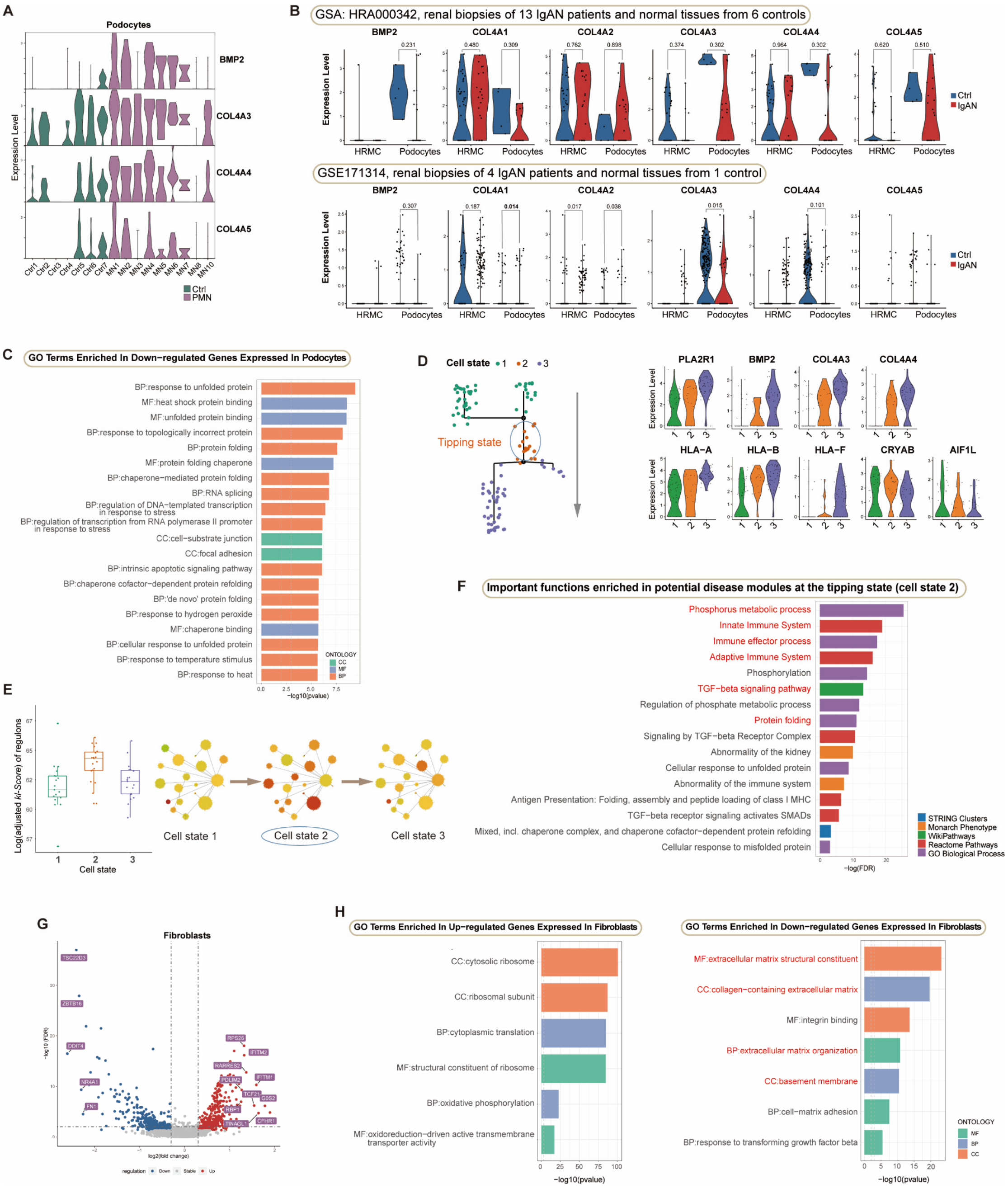

Supplemental Figure S3. Renal glomerular and interstitial cell alterations in PMN

A. Violin plot showing the expression levels of *BMP2*, *COL4A3*, *COL4A4*, and *COL4A5* in podocytes from each sample. **B.** Violin plots showing the expression levels of *BMP2*, *COL4A1, COL4A2, COL4A3*, *COL4A4*, and *COL4A5* in podocytes and HRMCs from datasets of HRA000342 and GSE171314. **C.** GO analysis presenting major downregulated pathways of podocytes. **D.** Three sub-states (sub-clusters) of 144 podocytes from PMN patients were inferred by trajectory analysis. Violin plot showing the expression levels of *PLA2R1, BMP2*, *COL4A3*, *COL4A4*, *HLA-A, HLA-B, HLA-F, CRYAB,* and *AIF1L* in podocytes across the three sub-states. **E.** Box plot of log(adjusted *KL-score*) of regulons for the three cell states, with the dynamical evolution of molecular network related to *BMP2* regulation in three states of podocytes during PMN progression. Gene modules in the network with darker and redder colors indicated a potential tipping point of PMN in cell state 2. **F**. Important functions enriched in potential disease modules at the tipping state, i.e., cell state 2. **G.**

Volcano plot showing major upregulated (red) and downregulated (blue) genes in fibroblasts. **H.** GO analysis presenting major up- and down-regulated pathways of fibroblasts.

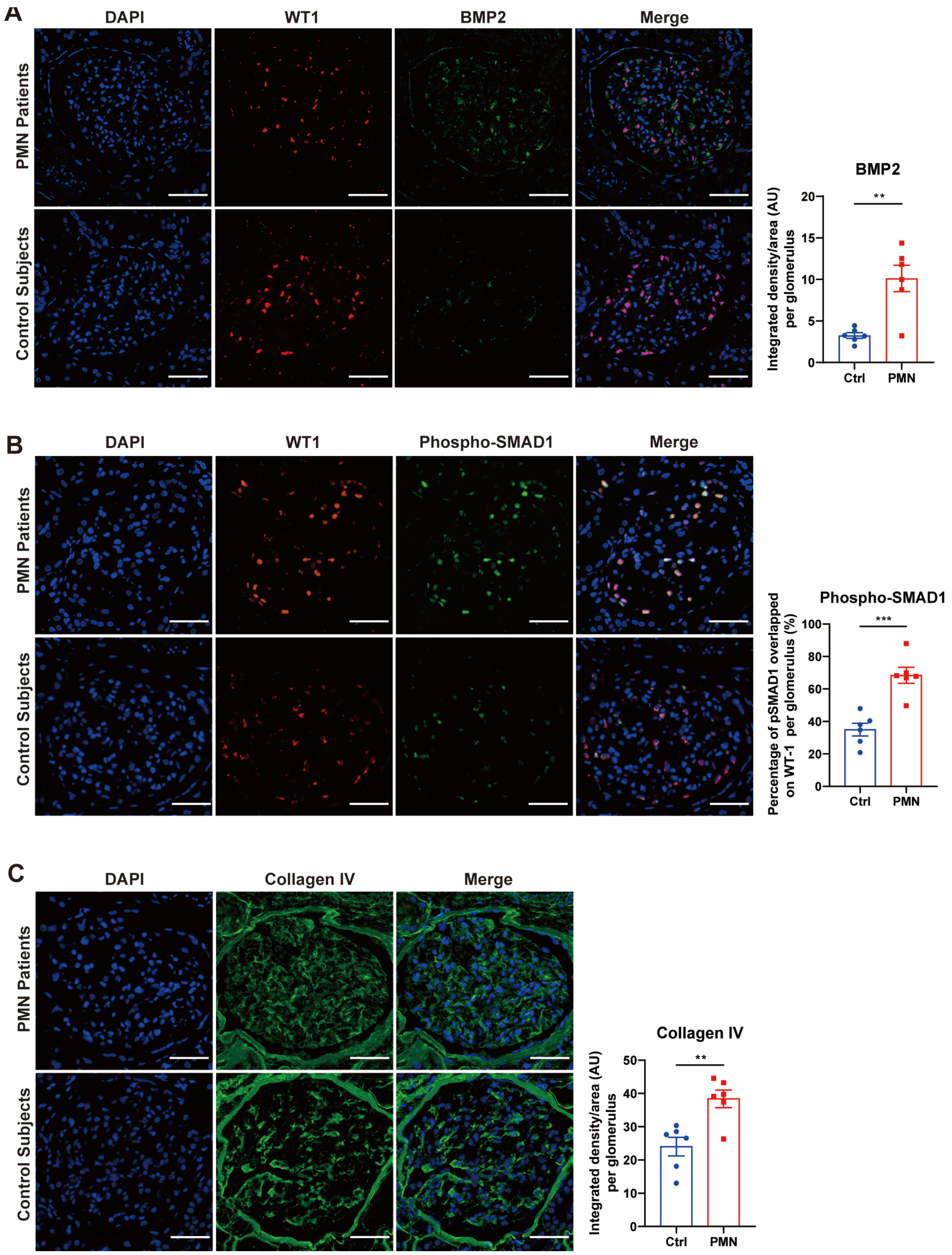

Supplemental Figure S4. Podocyte BMP2/pSMAD1/COL4 signaling in PMN patients outside of the scRNA-seq cohort

A. Immunofluorescence analysis of DAPI (blue), WT1 (red), and BMP2 (green) expression in paraffin-embedded kidney biopsy sections of PMN patients (upper) and control subjects (lower), with quantification of BMP2 fluorescence density per glomerulus. **B.** Immunofluorescence analysis of DAPI (blue), WT1 (red), and phosphorylated SMAD1 (green) expression in paraffin-embedded kidney biopsy sections of PMN patients (upper) and control subjects (lower), with quantification of the percentage of pSMAD1 and WT-1 double-positive cells per glomerulus. **C.** Immunofluorescence

analysis of DAPI (blue), and collagen IV (green) expression in paraffin-embedded kidney biopsy sections of PMN patients (upper) and control subjects (lower), with quantification of Collagen IV fluorescence density per glomerulus. For quantifications, each dot represents the average value from five random glomerular images from each patient/control.

, $P < 0.01$; ***, $P < 0.001$. Scale bar = 50 μm. N=6 per group for **A-C, and patients and controls were outside of the scRNA-seq cohort. Data are presented as mean ± SEM. Student's two-tailed t-test for **A-C**.

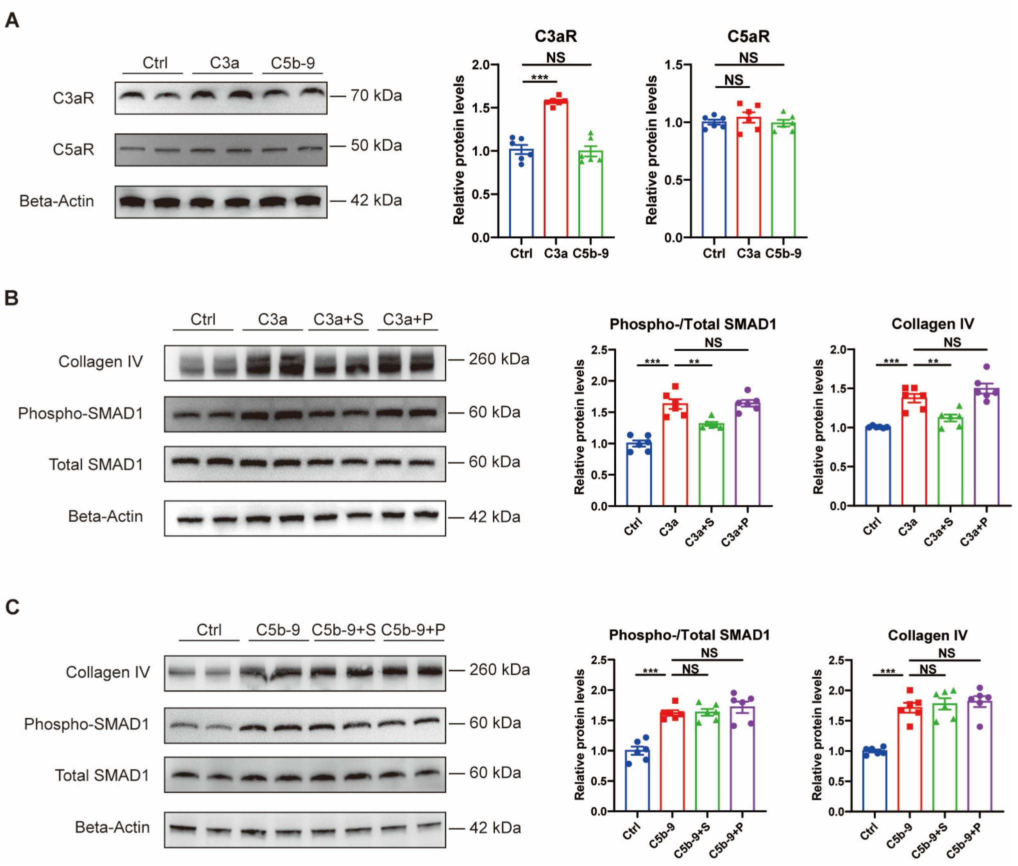

Supplemental Figure S5. Role of C3aR and C5aR in complement-induced BMP2/pSMAD1/COL4 signaling pathway

A. Western blot analysis showing the protein levels of C3aR and C5aR in podocyte cell lysates after incubation with C3a and C5b-9 for 48h, with the corresponding quantification. Beta-actin was used as the loading control. **B.** Western blot analysis showing the protein levels of collagen IV and phosphorylated and total SMAD1 in podocyte cell lysates after incubation with C3a, C3a+SB290157, and C3a+PMX-53 for 48h, with the corresponding quantification. Beta-actin was used as the loading control. **C.** Western blot analysis showing the protein levels of collagen IV and phosphorylated and total SMAD1 in podocyte cell lysates after incubation with C5b-9, C5b-9+SB290157, and C5b-9+PMX-53 for 48h, with the corresponding quantification. Beta-actin was used as the loading control.

, $P < 0.01$; ***, $P < 0.001$; NS, not significant. Each experiment was replicated for 5 times. N=6 per group. Data are presented as mean ± SEM. One-way ANOVA test for **A-C. S, SB290157; P, PMX-53.

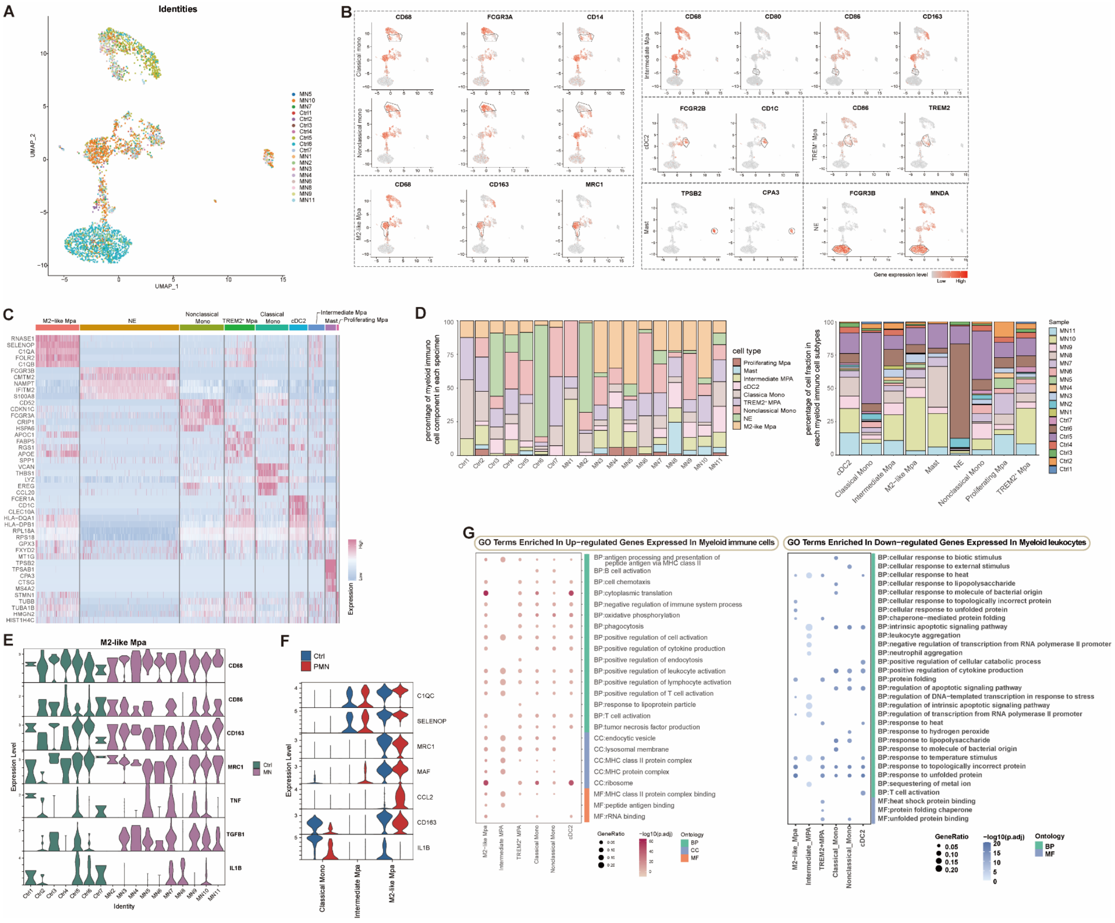

Supplemental Figure S6. Activation of M2-like macrophages in PMN

A. UMAP visualization of myeloid leukocytes from each sample. **B.** Expression distribution of marker genes of different myeloid leukocyte types. **C.** Heatmap indicating distinct gene expression patterns of different myeloid leukocyte types. **D.** Percentages of different myeloid leukocyte types from each sample. **E.** Violin plot showing the expression levels of macrophage polarization markers in M2-like macrophages from each sample. **F.** Expression level of marker and functional genes in classical monocytes, intermediate Mpa and M2-like Mpa, PMN versus Ctrl, shown in violin plots. **G.** GO enrichment presenting major upregulated and downregulated pathways of the monocyte/macrophage compartment.

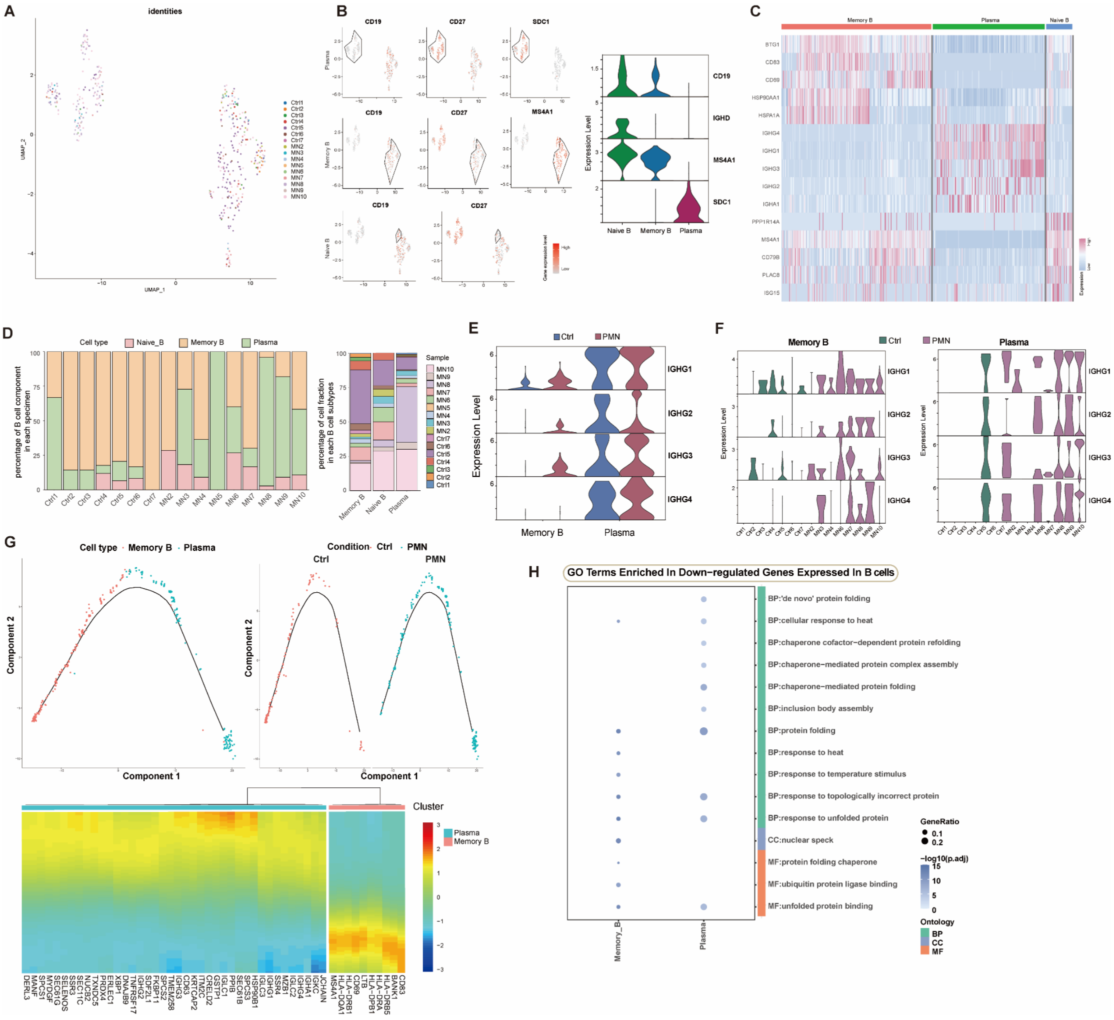

Supplemental Figure S7. Changes in memory B and plasma cells in PMN

A. UMAP visualization of B cells from each sample. **B.** Expression distribution of marker genes of different B cell types. **C.** Heatmap indicating distinct gene expression patterns of different B cell types. **D.** Percentages of different B cell types from each sample. **E.** Violin plot showing the expression levels of *IGHG1, IGHG2, IGHG3,* and *IGHG4* in memory B and plasma cells from PMN versus Ctrl. **F.** Violin plot showing the expression levels of *IGHG1, IGHG2, IGHG3,* and *IGHG4* in memory B and plasma cells from each sample. **G.** Pseudotime trajectory analysis presenting the differentiation trajectory from memory B cells to plasma cells (left) in PMN versus Ctrl (middle), with a heatmap indicating the expression of marker and functional genes (right). **H.** GO enrichment presenting major downregulated pathways of the B cells.

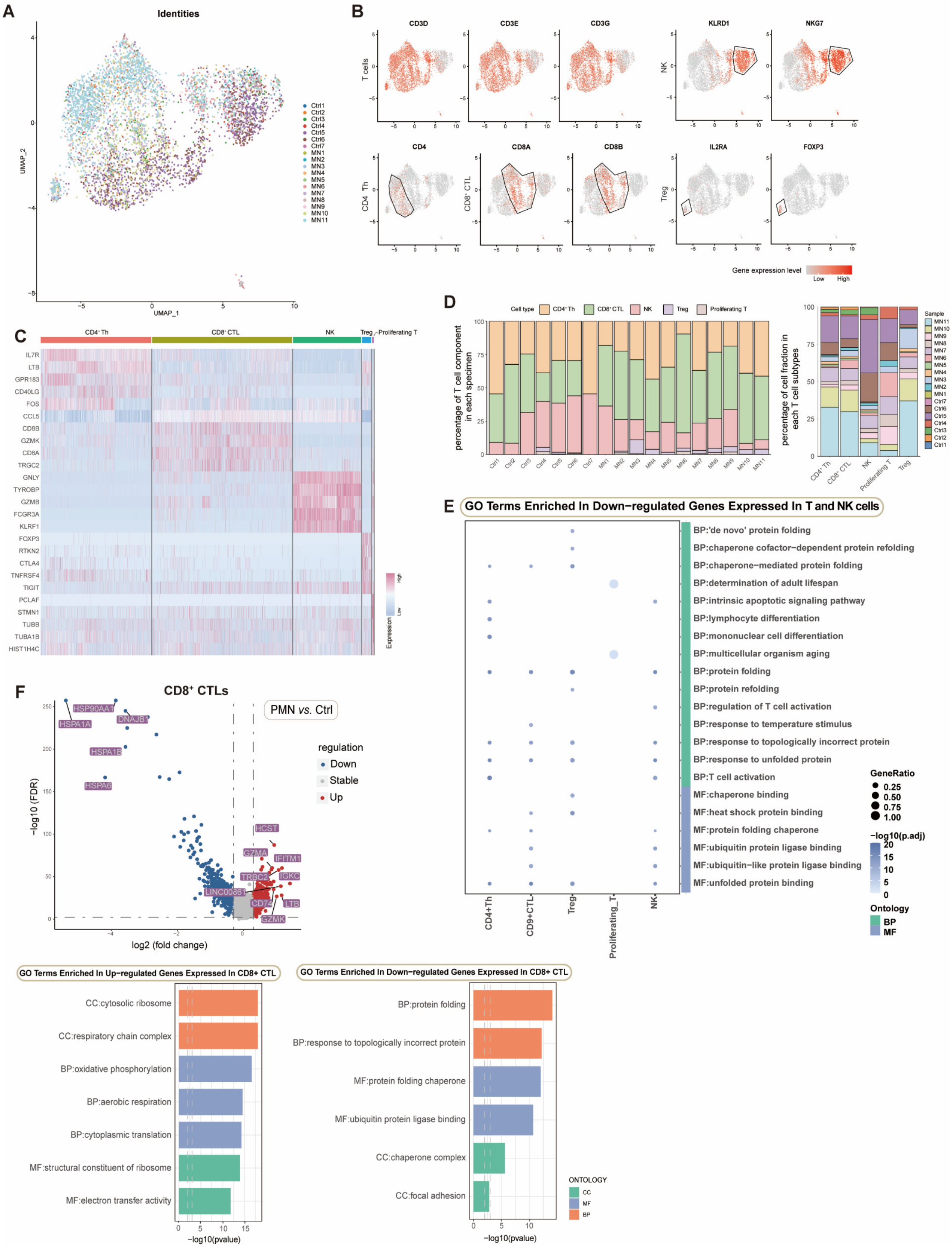

Supplemental Figure S8. Immunosuppressive trend of T lymphocytes in PMN

A. UMAP visualization of T and NK cells from each sample. **B.** Expression distribution of marker genes of different T and NK cell types. **C.** Heatmap indicating distinct gene expression patterns of different T and NK cell types. **D.** Percentages of different T and NK cell types from each sample. **E.** GO enrichment presenting major downregulated pathways of the T and NK cells. **F.** Volcano plot showing major upregulated (red) and downregulated (blue) genes in $CD8^+$ CTLs, with GO analysis presenting major up- and down-regulated pathways of $CD8^+$ CTLs.

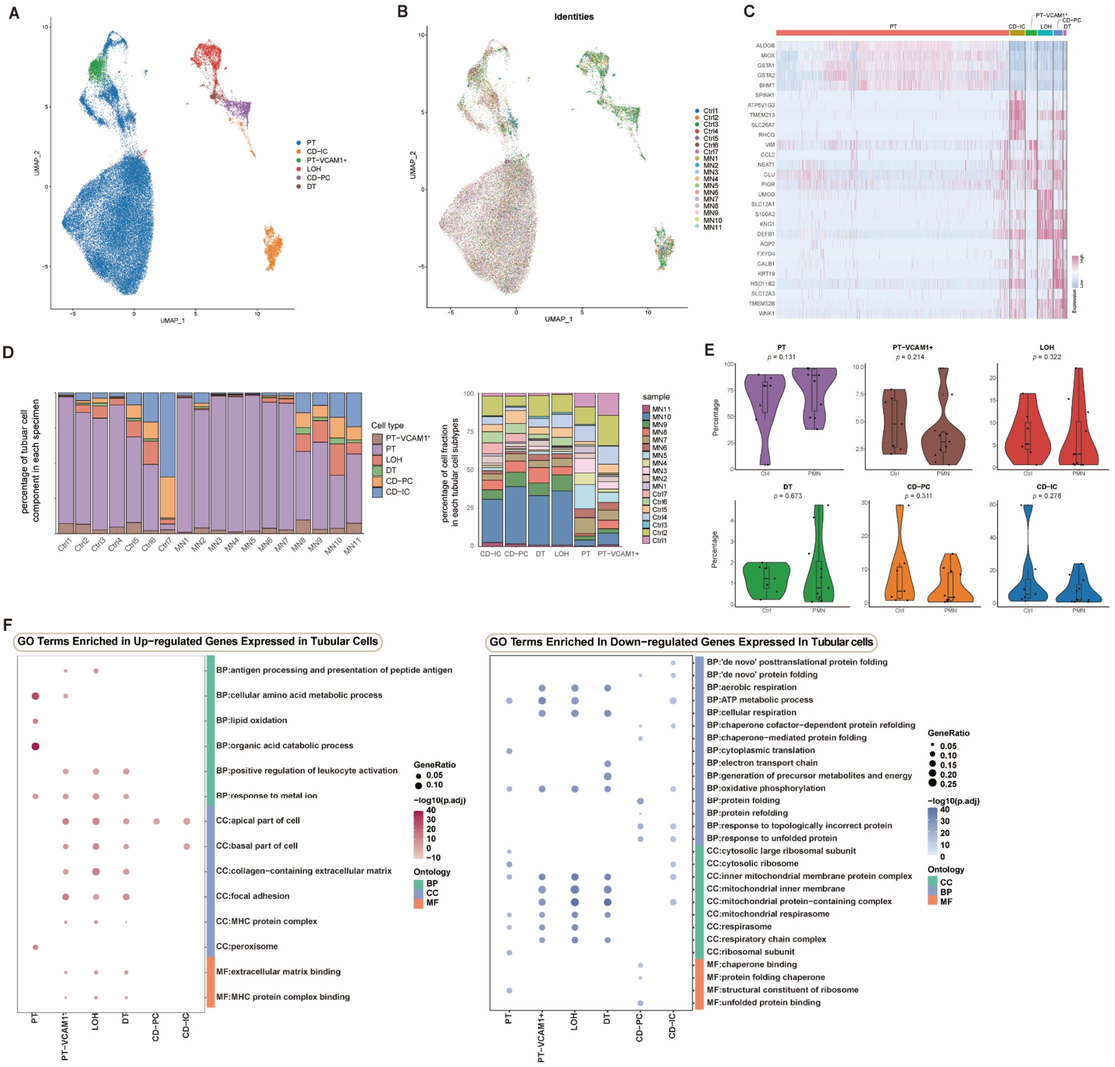

Supplemental Figure S9. Renal tubular cells in PMN

A. UMAP indicating subclustered tubular cells color-coded by different cell types. **B.** UMAP visualization of tubular cells from each sample. **C.** Heatmap indicating distinct gene expression patterns of different tubular cell types. **D.** Percentages of different tubular cell types from each sample. **E.** Respective cell percentages of different tubular cell types in PMN versus Ctrl. **F.** GO analysis presenting the major upregulated and downregulated pathways of different tubular cells.

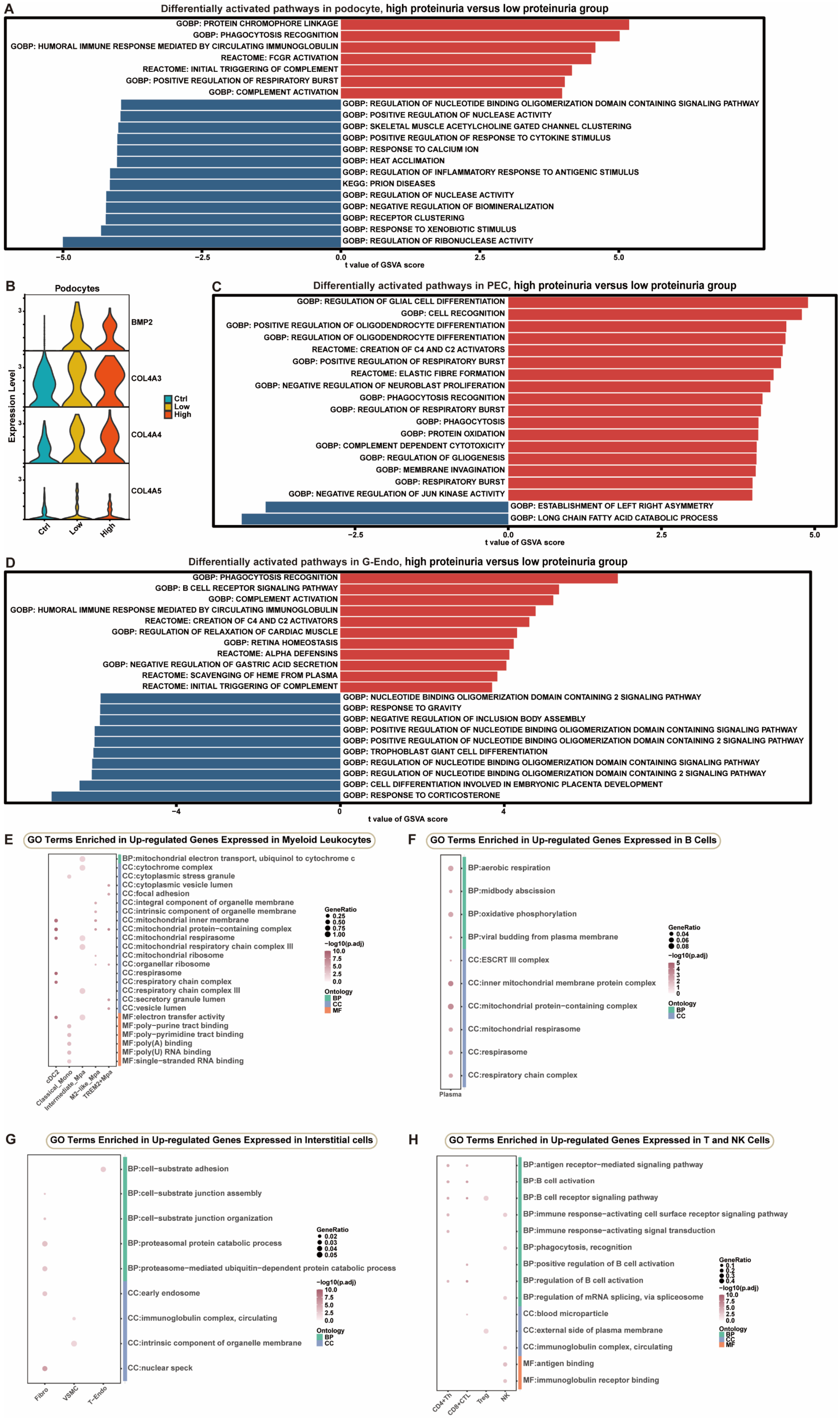

A
Differentially activated pathways in podocyte, high proteinuria versus low proteinuria group
GOBP: PROTEIN CHROMOPHORE LINKAGE
GOBP: PHAGOCYTOSIS RECOGNITION
GOBP: HUMORAL IMMUNE RESPONSE MEDIATED BY CIRCULATING IMMUNOGLOBULIN
REACTOME: FCGR ACTIVATION
REACTOME: INITIAL TRIGGERING OF COMPLEMENT
GOBP: POSITIVE REGULATION OF RESPIRATORY BURST
GOBP: COMPLEMENT ACTIVATION
GOBP: REGULATION OF NUCLEOTIDE BINDING OLIGOMERIZATION DOMAIN CONTAINING SIGNALING PATHWAY
GOBP: POSITIVE REGULATION OF NUCLEASE ACTIVITY
GOBP: SKELETAL MUSCLE ACETYLCHOLINE GATED CHANNEL CLUSTERING
GOBP: POSITIVE REGULATION OF RESPONSE TO CYTOKINE STIMULUS
GOBP: RESPONSE TO CALCIUM ION
GOBP: HEAT ACCLIMATION
GOBP: REGULATION OF INFLAMMATORY RESPONSE TO ANTIGENIC STIMULUS
KEGG: PRION DISEASES
GOBP: REGULATION OF NUCLEASE ACTIVITY
GOBP: NEGATIVE REGULATION OF BIOMINERALIZATION
GOBP: RECEPTOR CLUSTERING
GOBP: RESPONSE TO XENOBIOTIC STIMULUS
GOBP: REGULATION OF RIBONUCLEASE ACTIVITY
−5.0 −2.5 0.0 2.5 5.0
t value of GSVA score
B
Podocytes
BMP2
COL4A3
COL4A4
COL4A5
Expression Level
Ctrl
Low
High
C
Differentially activated pathways in PEC, high proteinuria versus low proteinuria group
GOBP: REGULATION OF GLIAL CELL DIFFERENTIATION
GOBP: CELL RECOGNITION
GOBP: POSITIVE REGULATION OF OLIGODENDROCYTE DIFFERENTIATION
GOBP: REGULATION OF OLIGODENDROCYTE DIFFERENTIATION
REACTOME: CREATION OF C4 AND C2 ACTIVATORS
GOBP: POSITIVE REGULATION OF RESPIRATORY BURST
REACTOME: ELASTIC FIBRE FORMATION
GOBP: NEGATIVE REGULATION OF NEUROBLAST PROLIFERATION
GOBP: PHAGOCYTOSIS RECOGNITION
GOBP: REGULATION OF RESPIRATORY BURST
GOBP: PHAGOCYTOSIS
GOBP: PROTEIN OXIDATION
GOBP: COMPLEMENT DEPENDENT CYTOTOXICITY
GOBP: REGULATION OF GLIOGENESIS
GOBP: MEMBRANE INVAGINATION
GOBP: RESPIRATORY BURST
GOBP: NEGATIVE REGULATION OF JUN KINASE ACTIVITY
GOBP: ESTABLISHMENT OF LEFT RIGHT ASYMMETRY
GOBP: LONG CHAIN FATTY ACID CATABOLIC PROCESS
−2.5 0.0 2.5 5.0
t value of GSVA score
D
Differentially activated pathways in G-Endo, high proteinuria versus low proteinuria group
GOBP: PHAGOCYTOSIS RECOGNITION
GOBP: B CELL RECEPTOR SIGNALING PATHWAY
GOBP: COMPLEMENT ACTIVATION
GOBP: HUMORAL IMMUNE RESPONSE MEDIATED BY CIRCULATING IMMUNOGLOBULIN
REACTOME: CREATION OF C4 AND C2 ACTIVATORS
GOBP: REGULATION OF RELAXATION OF CARDIAC MUSCLE
GOBP: RETINA HOMEOSTASIS
REACTOME: ALPHA DEFENSINS
GOBP: NEGATIVE REGULATION OF GASTRIC ACID SECRETION
REACTOME: SCAVENGING OF HEME FROM PLASMA
REACTOME: INITIAL TRIGGERING OF COMPLEMENT
GOBP: NUCLEOTIDE BINDING OLIGOMERIZATION DOMAIN CONTAINING 2 SIGNALING PATHWAY
GOBP: RESPONSE TO GRAVITY
GOBP: NEGATIVE REGULATION OF INCLUSION BODY ASSEMBLY
GOBP: POSITIVE REGULATION OF NUCLEOTIDE BINDING OLIGOMERIZATION DOMAIN CONTAINING SIGNALING PATHWAY
GOBP: POSITIVE REGULATION OF NUCLEOTIDE BINDING OLIGOMERIZATION DOMAIN CONTAINING 2 SIGNALING PATHWAY
GOBP: TROPHOBLAST GIANT CELL DIFFERENTIATION
GOBP: REGULATION OF NUCLEOTIDE BINDING OLIGOMERIZATION DOMAIN CONTAINING SIGNALING PATHWAY
GOBP: REGULATION OF NUCLEOTIDE BINDING OLIGOMERIZATION DOMAIN CONTAINING 2 SIGNALING PATHWAY
GOBP: CELL DIFFERENTIATION INVOLVED IN EMBRYONIC PLACENTA DEVELOPMENT
GOBP: RESPONSE TO CORTICOSTERONE
−4 0 4
t value of GSVA score
E
GO Terms Enriched in Up-regulated Genes Expressed in Myeloid Leukocytes
BP:mitochondrial electron transport, ubiquinol to cytochrome c
CC:cytochrome complex
CC:cytoplasmic stress granule
CC:cytoplasmic vesicle lumen
CC:focal adhesion
CC:integral component of organelle membrane
CC:intrinsic component of organelle membrane
CC:mitochondrial inner membrane
CC:mitochondrial protein−containing complex
CC:mitochondrial respirasome
CC:mitochondrial respiratory chain complex III
CC:mitochondrial ribosome
CC:organellar ribosome
CC:respirasome
CC:respiratory chain complex
CC:respiratory chain complex III
CC:secretory granule lumen
CC:vesicle lumen
MF:electron transfer activity
MF:poly−purine tract binding
MF:poly−pyrimidine tract binding
MF:poly(A) binding
MF:poly(U) RNA binding
MF:single−stranded RNA binding
cDC2
Classical_Mono
Intermediate_Mpa
M2−like_Mpa
TREM2+Mpa
GeneRatio
0.25
0.50
0.75
1.00
−log10(p.adj)
10.0
7.5
5.0
2.5
0.0
Ontology
BP
CC
MF
F
GO Terms Enriched in Up-regulated Genes Expressed in B Cells
BP:aerobic respiration
BP:midbody abscission
BP:oxidative phosphorylation
BP:viral budding from plasma membrane
CC:ESCRT III complex
CC:inner mitochondrial membrane protein complex
CC:mitochondrial protein−containing complex
CC:mitochondrial respirasome
CC:respirasome
CC:respiratory chain complex
Plasma
GeneRatio
0.04
0.06
0.08
−log10(p.adj)
5
4
3
2
1
0
Ontology
BP
CC
G
GO Terms Enriched in Up-regulated Genes Expressed in Interstitial cells
BP:cell−substrate adhesion
BP:cell−substrate junction assembly
BP:cell−substrate junction organization
BP:proteasomal protein catabolic process
BP:proteasome−mediated ubiquitin−dependent protein catabolic process
CC:early endosome
CC:immunoglobulin complex, circulating
CC:intrinsic component of organelle membrane
CC:nuclear speck
Fibro
VSMC
T−Endo
GeneRatio
0.02
0.03
0.04
0.05
−log10(p.adj)
10.0
7.5
5.0
2.5
0.0
Ontology
BP
CC
H
GO Terms Enriched in Up-regulated Genes Expressed in T and NK Cells
BP:antigen receptor−mediated signaling pathway
BP:B cell activation
BP:B cell receptor signaling pathway
BP:immune response−activating cell surface receptor signaling pathway
BP:immune response−activating signal transduction
BP:phagocytosis, recognition
BP:positive regulation of B cell activation
BP:regulation of B cell activation
BP:regulation of mRNA splicing, via spliceosome
CC:blood microparticle
CC:external side of plasma membrane
CC:immunoglobulin complex, circulating
MF:antigen binding
MF:immunoglobulin receptor binding
CD4+Th
CD8+CTL
Treg
NK
GeneRatio
0.1
0.2
0.3
0.4
−log10(p.adj)
10.0
7.5
5.0
2.5
0.0
Ontology
BP
CC
MF

Supplemental Figure S10. Comparison between PMN patients with low and high proteinuria levels
A. Major upregulated (red) and downregulated (blue) pathways of podocytes in PMN patients with high versus low proteinuria levels, according to GSVA. **B.** Violin plot showing the expression levels of *BMP2*, *COL4A3*, *COL4A4*, and *COL4A5* in podocytes from 3 groups. **C.** Major upregulated (red) and downregulated (blue) pathways of PECs in PMN patients with high versus low proteinuria levels, according to GSVA. **D.** Major upregulated (red) and downregulated (blue) pathways of G-Endos in PMN patients with high versus low proteinuria levels, according to GSVA. **E.** GO analysis presenting major upregulated pathways of myeloid leukocytes. **F.** GO analysis presenting major upregulated pathways of B cells. **G.** GO analysis presenting major upregulated pathways of renal interstitial cells. **H.** GO analysis presenting major upregulated pathways of T and NK cells.
